# How Fire History, Fire Suppression Practices and Climate Change Affect Wildfire Regimes in Mediterranean Landscapes

**DOI:** 10.1371/journal.pone.0062392

**Published:** 2013-05-02

**Authors:** Lluís Brotons, Núria Aquilué, Miquel de Cáceres, Marie-Josée Fortin, Andrew Fall

**Affiliations:** 1 Grup d’Ecologia del Paisatge, Àrea de Biodiversitat, CTFC (Centre Tecnològic Forestal de Catalunya), Solsona, Catalonia, Spain; 2 CREAF (Centre de Recerca Ecològica i Aplicacions Forestals), Bellaterra, Spain; 3 Department of Ecology & Evolutionary Biology, University of Toronto, Toronto, Ontario, Canada; 4 Resource and Environmental Management, Simon Fraser University and Gowlland Technologies Ltd, Lasqueti, British Columbia, Canada; The Ohio State University, United States of America

## Abstract

Available data show that future changes in global change drivers may lead to an increasing impact of fires on terrestrial ecosystems worldwide. Yet, fire regime changes in highly humanised fire-prone regions are difficult to predict because fire effects may be heavily mediated by human activities We investigated the role of fire suppression strategies in synergy with climate change on the resulting fire regimes in Catalonia (north-eastern Spain). We used a spatially-explicit fire-succession model at the landscape level to test whether the use of different firefighting opportunities related to observed reductions in fire spread rates and effective fire sizes, and hence changes in the fire regime. We calibrated this model with data from a period with weak firefighting and later assess the potential for suppression strategies to modify fire regimes expected under different levels of climate change. When comparing simulations with observed fire statistics from an eleven-year period with firefighting strategies in place, our results showed that, at least in two of the three sub-regions analysed, the observed fire regime could not be reproduced unless taking into account the effects of fire suppression. Fire regime descriptors were highly dependent on climate change scenarios, with a general trend, under baseline scenarios without fire suppression, to large-scale increases in area burnt. Fire suppression strategies had a strong capacity to compensate for climate change effects. However, strong active fire suppression was necessary to accomplish such compensation, while more opportunistic fire suppression strategies derived from recent fire history only had a variable, but generally weak, potential for compensation of enhanced fire impacts under climate change. The concept of fire regime in the Mediterranean is probably better interpreted as a highly dynamic process in which the main determinants of fire are rapidly modified by changes in landscape, climate and socioeconomic factors such as fire suppression strategies.

## Introduction

Fire is a key disturbance in many terrestrial ecosystems [1]. Current available data show that future trends in fire drivers, such as climate warming or land use changes, may lead to an increasing impact of fires on ecosystems worldwide with unknown effects on biodiversity patterns and ecosystem services [2], [3]. Changes in fire regimes associated with new land use for human activities may lead to large scale shifts in vegetation types [4], [5]. Understanding the role and the relative weight of different factors leading to changes in fire regimes is thus of critical importance to anticipate the fate of biodiversity or to implement management strategies aiming at mitigating or modulating the impact of fires arising from such changes.

Fire regimes are determined by complex interactions between climate, land use, vegetation attributes and the pattern of ignition [6]–[8]. Different factors have been hypothesised to drive fire regimes at different spatial scales [2]. At small spatial and temporal scales, the amount and continuity of fuel as well as the number and spatial distribution of ignitions have been shown to determine the number of fires and their size [6], [9], [10]. However, at larger temporal and spatial scales, fire regimes appear to be more determined by climatic variability with short periods of high fire risk linked to particular weather conditions accounting for most fire events [11]. At present, the relative contribution of fuel load and vegetation composition at a landscape scale versus climate forcing and the distribution of fire ignitions is under debate and appears to be context dependent even within a given area [12], [13]. There is the concern that climate change may rapidly alter these conditions in many regions [7], [14] and reinforce the role of climate as a determinant of fire impacts, favouring climate driven fire regimes [2], [15].

As fire events do not only impact ecological communities but also have major negative effects on human activities, several agencies have devoted considerable efforts to suppress fires [16], [17]. Fire suppression is a direct anthropogenic activity altering a fire regime, even though its significance has been a point of debate [18], [19]. Fire suppression efforts are aimed at limiting fire impact by decreasing fire severity or decreasing effective fire size. There is some evidence that fire suppression reduces the number and frequency of large fires in several regions from sub-boreal and boreal forests in Canada [20] to the Mediterranean region [16], [21]. At the same time, available evidence also suggests that, in the long-term, intense fire suppression may result in larger than normal fires because of fuel build-up [22]. In regions where firefighting policies are implemented, fire regimes are likely to differ from those dominating natural forest systems [16], [23]. In these cases, fire regimes may be especially difficult to characterise and become highly dynamic in nature, leading to formidable challenges in the prediction of fire effects [12], [24]. The understanding of the different contributions of these factors to landscape change is of critical interest [25], especially if predictions of further change areto be anticipated and consequences for fire regimes understood [26]. Future projections of fire effects at the landscape scale rely on the understanding of the causes behind short-term fire regime changes. It is therefore important to develop tools for assessing expected effects of wildfires under different scenarios of climate conditions, fire suppression strategies and land use changes.

Very few studies have investigated the potential contrasting effects of climate change and fire suppression efforts on fire impact at the landscape scale [27], [28]. In addition, it is yet uncertain whether and how landscape history should be included in the planning of fire suppression strategies. Here, we use a Mediterranean region as a case study and assess, in a context of climate change, the role of fire suppression in determining essential fire regime attributes such as the amount of area burnt per year and the percentage of area burnt by large fires.

Our premise is that improved fire-behaviour knowledge by fire brigades may currently constrain the occurrence of large fires and strongly influence the effective area burnt in the study area [29]. We first calibrated and validated a landscape fire succession model with observed fire regime data under conditions of low firefighting effectiveness. Then, to determine what levels of firefighting effectiveness are needed to appropriately reproduce recently observed fire regime statistics, we ran landscape model simulations under different fire suppression strategies while controlling for other major determinants of fire regime (ignition pattern, climate variability and land use pattern). In these simulations, we also wanted to determine the degree to which firefighting could be benefiting from the use of recent burnt areas with low fuel availability as key opportunities for fire suppression. Finally, we conducted another simulation exercise to determine the potential role of current firefighting strategies in constraining fire regimes in the near future, under a context of likely increase in the frequency of climatically adverse years.

## Study Area

The study area was Catalonia a region located in the north-eastern corner of Spain). Catalonia is extensively covered with shrubland and forests (about 60%) but human presence since pre-historical times has led to large-scale changes in species composition and distribution of dominant species in historical and recent times. The majority of the study area has a Mediterranean climate, with winter precipitation and summer drought [30]. According to the Catalan land-cover map, shrublands cover 36.7% of the total wild-land area of 1,950,326 ha, with a diverse specific and mainly evergreen composition [31]. Forest composition as described by the First Ecological Forest Inventory of Catalonia [32] shows that conifers occur in 60.3% of the total forested area (20% *Pinus halepensis*, 18.4% *Pinus sylvestris*, 11.7% *Pinus nigra*), while sclerophilous and deciduous species cover the remaining 39.7% (15.4 *Quercus ilex*, 7% *Quercus faginea*, 5.3% *Quercus suber*).

**Figure 1:**
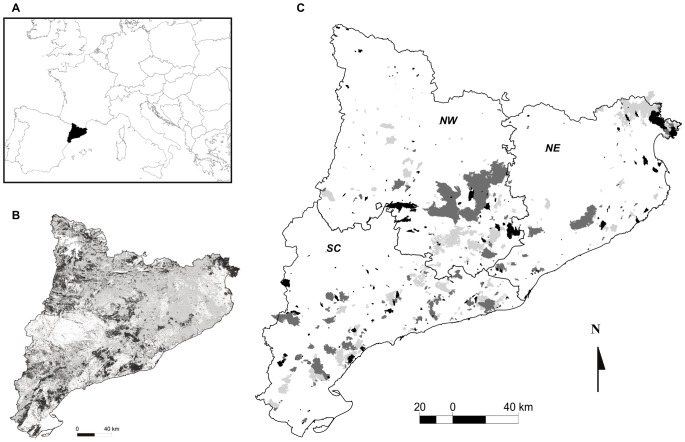
Location of Catalonia in the European context (A). Dynamic land cover types in the year 2000 (B, see Appendix S2 for further information) with forest areas (in light grey) and shrublands (in dark grey). Representation of wildfires that occurred between 1975 and 1988 (light grey), between 1989 and 1999 (dark grey) and wildfires occurred between 2000 and 2010 (black) (C). Following [36], Catalonia is divided in three bioclimatic regions: North-West (NW), North-East (NE) and South-Central (SC).

Fire is a major landscape driver in the region, with about 25% of the wild-land area (about 340,000 ha) being burnt between 1975 and 2010 (Figure 1). Stand-replacing fires appear to be the most common in Mediterranean vegetation [33]. A trend towards larger fires has been observed for Catalonia during the second half of the 20th century [33], [34]. In fact Pausas & Fernández-Muñoz [12] found historical changes in fire regimes in the area, with a higher incidence of drought-driven fires after the 1970’s and a pre-eminence of fuel-limited fires before the 1970’s. Wildfires heterogeneously influenced forest landscapes in the study area, causing stronger impacts in southern-coastal and central sub-regions with warmer Mediterranean and continental climates than in northern-coastal sub-regions with a stronger wind impact [35]. Therefore, we used the bioclimatic sub-regions identified by [36] to account for the role of landscape context and climatic gradients on fire regimes. In addition, available data suggest that the climatic conditions leading to adverse fire prone summers have been increasing the last years and projections of future climate change indicate that the number of days per summer with fire-prone conditions will continue to increase [30].

In recent times, increased resource investment and efficiency in firefighting activity has lead to most of the fires being controlled at early stages in their development, especially near highly populated areas [22]. While firefighting efforts before the year 1999 had traditionally focussed on vigilance and early detection of ignitions, key enhancements of firefighting capacity involve the introduction, after 1999, of logical analyses of fire behaviour. This knowledge allows technical fire brigades to anticipate changes in fire propagation and efficiently use controlled fires during extinction [29]. When anticipated and included in firefighting strategies, discontinuities in fuel distribution hinder fire propagation and create favourable opportunities for stopping fires [37]. These opportunities may be related to topography, vegetation heterogeneity or landscape history such as fire scars leading to sharp decreases in fuel loads and continuity at the landscape scale. We therefore have identified two periods according to the overall fire-suppressing effectiveness in the study region with the pre-2000 period described as low fire-fighting capability, and the post-2000 period with high fire-fighting capability expected to lead to decreases in annual burnt area and a lower impact of large fires.

## MEDFIRE Model

**Figure 2:**
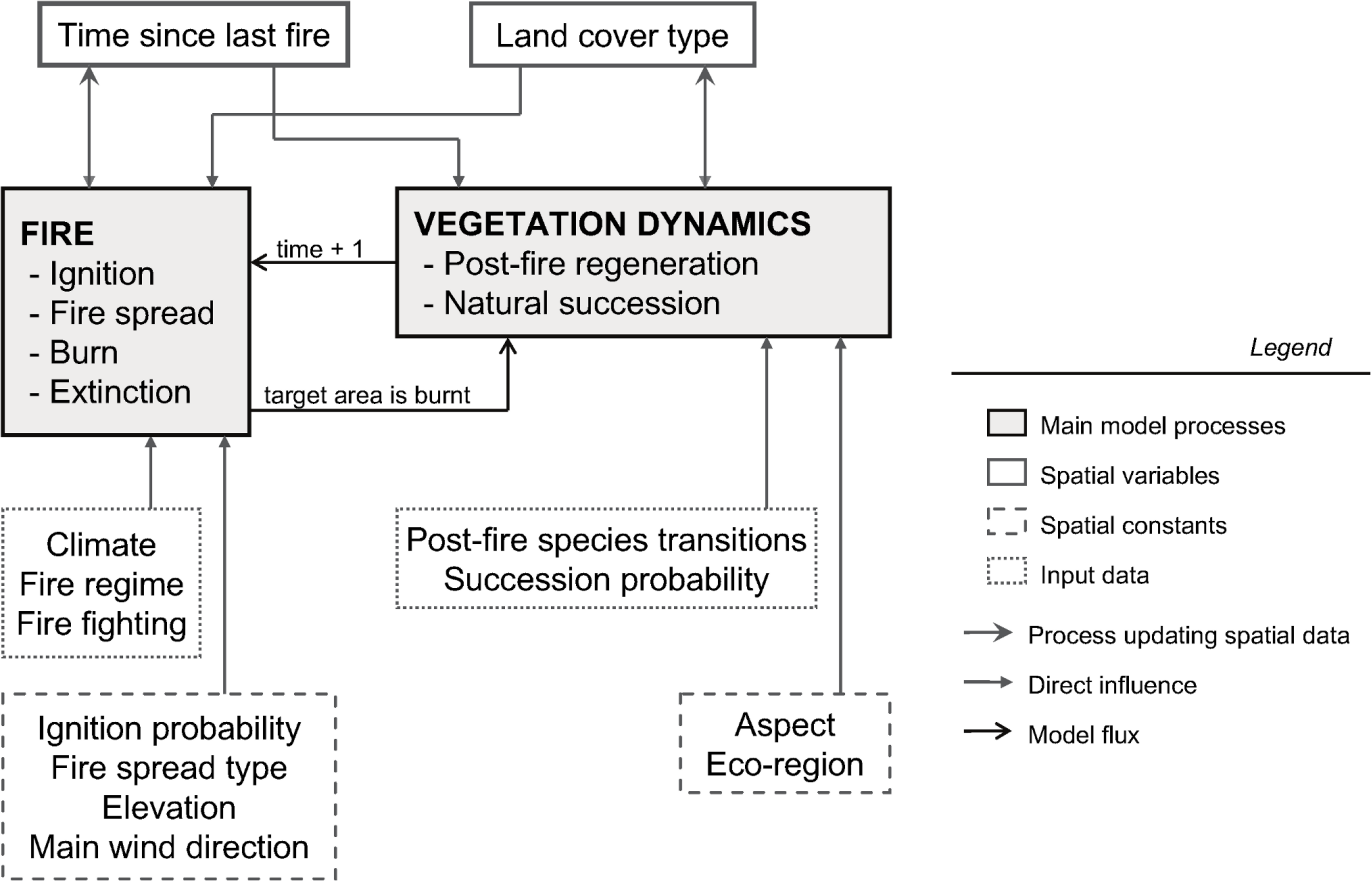
Conceptual design of the MEDFIRE model. *Land cover type* and *time since last fire* are state variables. The fire sub-model is responsible for updating *time since last fire*, whereas *land cover type* is updated in the vegetation dynamics sub-model. Fire processes occur sequentially until the annual target area is burnt. After that, the vegetation dynamics sub-model takes place to complete the annual cycle.

The MEDFIRE model is a novel spatially explicit stochastic model that simulates landscape composition changes derived from vegetation dynamics and wildfire disturbances in a Mediterranean context. The model simulates the primary processes of a landscape fire-succession model (Figure 2) [38]: vegetation maturation and succession, fire ignition, fire spread and post-fire effects (vegetation transitions after fire). In addition, the model allows mimicking fire suppression actions that directly affect the simulated fire regime. The MEDFIRE model shares many characteristics with other landscape simulation tools accounting for spatial interactions, such as the LANDIS model [39], because multiple processes are simulated iteratively in a spatial raster framework for fixed time discrete steps. Similar to other landscape models (e.g., the Mauricie Model [40] and the Vermillion Landscape Model [41]), the MEDFIRE model was implemented using the spatio-temporal modeling tool SELES [42]. We follow the updated Overview, Design concepts, and Details (ODD) protocol of [43] to describe the MEDFIRE model in further detail.

### Model Overview

#### Purpose

The MEDFIRE model aims to examine the spatial interaction between wildfires and vegetation dynamics in heterogeneous landscapes. It has been designed to model different fire regime drivers to allow the investigation of their relative effects on the resulting annual area burnt distribution, fire size distribution and landscape composition at short- and medium-term time scales in a Mediterranean context. The model permits the characterization of the spatial variation in burning and land cover changes under different climatic scenarios and fire suppression strategies. The MEDFIRE model assumes that the main driver of the fire regime in the study area is climate [6], [12], [30]. Fire regime features, such as fire size and total area burnt, are initially dictated by climatic conditions but can be modulated by fire suppression strategies and landscape and vegetation features (Figure 2).

#### State variables and scale

State variables in the MEDFIRE model are spatial variables that describe the landscape context and conditions. They are represented in raster format and cover the full extent of the study area at 100 m resolution. The temporal scale is fixed and one time step represents one year; simulations are normally run for a few decades.

The state variables whose values change as a result of spatial processes are *land cover type* (LCT) and *time since last fire* (TSF). LCT is a categorical variable whose states are divided into 13 land covers that can be affected by fire disturbance because of their burnable condition (including forests, shrublands, croplands and grasslands), and three land covers that cannot (urban, water and rocks). Although several LCTs are burnable, and therefore contribute to fire spread, only shrublands and forests can undergo land cover changes. This approach may constrain the application of the MEDFIRE model in cases in which land abandonment or urbanization are widespread and rapid. Yet, we consider that the model is adequate in situations in which such land use changes are of minor importance compared to fire and forest maturation in determining landscape changes over the study time frame [36]. Forested cells include information of the dominant tree species in the canopy. The list of tree species considered (*Pinus halepensis*, *Pinus nigra*, *Pinus pinea*, *Pinus sylvestris*, *Quercus ilex* and *Quercus suber*) is representative of the Mediterranean landscapes dominant in Catalonia and other areas of the Western-Mediterranean [4], [32]. Three additional categories allow completing the classification of forests in the study area: other conifers, other *Quercus* species and other trees. TSF is an integer variable that is used as a surrogate of vegetation age and of timber volume, for cells belonging to shrubland and forest cover types only.

Other spatial state variables describe additional landscape features but are static in the current version of the model: ignition probability, bioclimatic region, fire spread type, elevation, aspect and main wind direction (Appendix S1). The ignition probability layer is used to stochastically determine the spatial location of new fires while taking into account the main ignition factors at the local scale (Appendix S1). The bioclimatic region layer was introduced mainly to allow the regionalization of post-fire vegetation transitions. The spread type layer describes the proportion of wind-driven versus topography-driven fires in each cell. Main wind direction accounts for the spread speed in wind-driven fires, whereas elevation and aspect are used to determine spread rate in fires driven by topography.

#### Process overview and scheduling

Fire disturbance and vegetation change processes are designed as two separate sub-models each of whose action needs to be completed before the next one starts. Each one-year time step, the fire disturbance sub-model is scheduled first, followed by the sub-module responsible for vegetation changes. The fire sub-model begins by setting the potential total area to be burnt. The sub-model simulates fires sequentially until the potential total area to be burnt is reached (Appendix S1). For each fire, the model first chooses a potential size and an ignition location. The location chosen for ignition is used to determine the spread type (relief- or wind-driven) and assumes that when a fire is driven by wind this factor overrides the modulating effects of topography (Appendix S1). If fire suppression is not active, the fire is allowed to spread until the potential fire size is attained. However, if fire suppression is active not all the cells potentially affected by a fire will be effectively burnt. The fire sub-model resets the value of time since fire to zero each time a given cell is effectively burnt. The vegetation dynamics sub-model iterates through all cells of the grid and updates the LCT of a given cell in the following two cases: (1) if the cell was burnt by the fire sub-module, its LCT may change according to a set of post-fire vegetation transition probabilities; (2) if the cell was not burnt but its LCT is shrubland, then natural succession from shrubland to forest may occur. Our present use of the MEDFIRE model is limited to time horizons of only a few decennia, so we are not interested in seral transitions between forest species, although such dynamics could be straightforwardly implemented in future versions of the model. Reporting tasks are carried out at the end of each time step. Detailed sub-model descriptions, including specific formal procedures and parameterization, can be found in Appendix S1 and Appendix S2.

### Design Concepts

#### Fire regime

The fire sub-model has been designed to allow the fire spread rate to partially depend on the main factors determining fire shapes in real landscapes [6], [30]. More specifically, fire spread rate is calculated as a function of fuel load (using time since fire as a proxy), topography or wind direction and land cover category (Appendix S1). The shape of a fire arises as a result of distinct rates of fire spread from one cell to adjacent cells. In contrast, fire size is primarily determined by applying a top-down approach in which the potential area to be burnt is chosen from an input statistical distribution of fire sizes [44]. Different fire size distributions are used depending on the climatic severity of the year. Adverse years are characterized by a high number of weather risk days [30], [45]. Therefore, the distribution of fire sizes corresponding to adverse years specifies a higher proportion of large wildfires compared to the distribution corresponding to normal (non-adverse) years [45]. The potential total area to be burnt is also drawn from a statistical distribution that differs between adverse and normal years.

**Figure 3:**
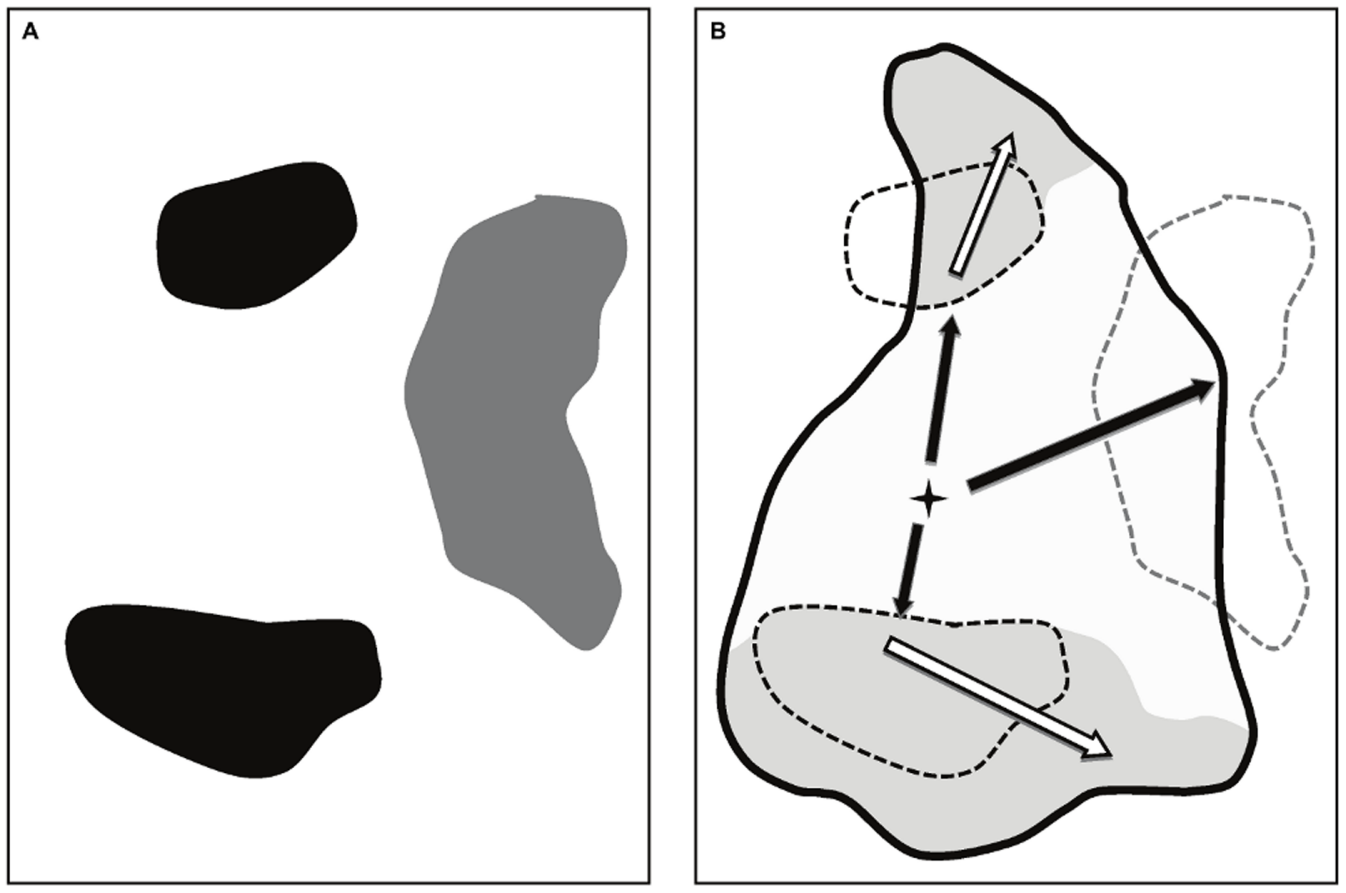
Description of the effects of opportunistic fire suppression on effective fire size. (A) Historic fires in a region, where black patches show recent burns with time since last fire values lower than 15 years and grey patches correspond to older fires. (B) Fire spread of a new simulated fire in the area. Potential target area (black thick line) is larger than the effective area burnt (white filling within the target area) because of opportunistic firefighting opportunities generated by recent fires in (A). Suppressed areas are shown in grey and main spread axes are shown in arrows. Spread occurring within effective area burnt (black arrows) and potentially, within the suppressed area (white arrows) is shown.

The explicit inclusion of processes leading to fire extinction may help in gaining insight into the factors determining the effective fire regime [46]. In the MEDFIRE model, two distinct fire suppression strategies are implemented, both related to the concept of firefighting opportunity, which is defined as an instance in which low fire intensity allows fire fighters to control first and ultimately extinguish the fire front. The first suppression strategy (here called *active suppression*) concerns opportunities generated in areas where spread rate (an indicator of fire intensity) is low enough to allow firefighting crews to stop a fire from spreading further. The second suppression strategy (here called *opportunistic suppression*) is based on opportunities derived from recent burned areas (fire scars). Previous detailed knowledge of the location and low fuel loads in these areas is assumed to lead to a significant increase in firefighting capacity. The two suppression strategies differ in the mechanisms driving such reductions in effective area burnt: while the opportunistic suppression strategy takes advantage of opportunities derived from past fire history (Figure 3), active fire suppression mimics overall firefighting capacity under slow spread conditions.

We have distinguished these two strategies because, while active fire suppression effectiveness may depend on the amount of resources allocated to firefighting, the effectiveness of opportunistic fire suppression is in fact mainly determined by historical fire patterns and does not require high-cost firefighting techniques or strategies. Therefore, it is important to identify to which degree the impact of fire suppression may be explained by the interaction with previous fire history, or may be due to an increase in funding or firefighting skills. Both suppression strategies lead to an effective fire size that is smaller than the potential fire size. As a consequence, the effective total area burnt and distribution of fire sizes are important emergent properties of the MEDFIRE model. These properties allow assessing the relative importance of climatic variability (i.e. proportion of climatically adverse years) and fire suppression strategies in determining the fire regime and its impacts.

#### Vegetation dynamics

In MEDFIRE, forests are simply described using the dominant tree species for each grid cell (Appendix S1). Therefore, the model cannot handle the complexity derived from heterogeneity within forest stands: spatial interactions between individuals of the same or different species are not considered [47]. Moreover, the long-term successional replacement of one dominant tree species by another is also not considered, due to the focus of the model on short- to medium-term time periods. Thus, the dominant tree species in a forest can only change after the impact of fire disturbance. Post-fire transitions in dominant species are implemented according to two approaches: non-spatial stochastic transitions or neighborhood species contagion. In the first case, the new cover class is chosen using a multinomial distribution with transition probabilities that depend on the pre-fire cover class as well as on other factors such as aspect, the bioclimatic region and whether the cell has been burnt in preceding years [4]. In the second case, the new cover class is chosen among those neighbors that also burnt in the current year and shared the same pre-fire cover class. New forest areas can arise from shrublands through succession: shrubland areas not recently burnt may become forests depending on the availability of mature forests among neighboring cells. Forest is assumed to be in a relatively stable state; once forest is present in a cell it does not change to other forest types unless it burns.

### Model Details

#### Initialization, input data and sub-model description

Initial LCT values were obtained using spatially explicit information on tree species distribution in Catalonia from the Spanish Forest Map and forest compositional data derived from forest inventories [48]. Complementary information on non-forest categories was obtained from the Land Cover Maps of Catalonia [31]. Initial TSF values were derived from a subset of available information on wildfire spatial distribution and fire historical statistics in the region (1975–2010 fires) including information on wildfires greater than 50 ha. See online Appendix S2 for further details on data sources and specific information about the processes employed to initialize dynamic and static spatial variables of the MEDFIRE model.

Available fire statistics for the 1975–1988 period were used to build input distributions for potential total area burnt and potential fire sizes. We differentiated between adverse and normal years of the 1975–1988 period using meteorological data. Spread type layers were based on [49]. Post-fire transition probabilities for vegetation regeneration by dominant tree species were based on [4]. Specific parameter values used in all modelled processes can be found in Appendix S2. Unless specified, the initialization for all the simulations described below was the same in terms of initial spatial conditions and parameters.

## Methods

### Validation of the MEDFIRE Model

We used available fire statistics for the period between 1989 and 1999 to validate fire regime descriptors generated by the MEDFIRE model. This period precedes the implementation in the year 2000 of key enhancements in Catalonia in fire fighting capacity involving the introduction of logical analyses of fire behaviour [29]. As fire regime descriptors, we used the total amount of area burnt and the relative importance of large fires, measured as the percentage of area burnt by large fires (those larger than 500 ha). We therefore expect that the model calibrated in the 1975–1988 period would be able to reproduce overall burnt area and the contribution of large fires to the fire regime during this period.

We ran 100 simulation replicates of the MEDFIRE model for this eleven-year period using meteorological records of summer weather conditions to determine adverse (4 years) and non-adverse years (7 years) in the model. To determine whether the modelled fire regime was compatible with observed data we compared the observed fire regime descriptor (either total amount of area burnt or the percentage of large fires) with the corresponding interval containing 95% of simulated values. Given the spatial variation in fire regimes known to exist in Catalonia [35], we also determined the compatibility of the simulated fire regime with the two observed fire regime descriptors for each of the three bioclimatic sub-regions shown in Figure 1.

### Reproducibility of the Fire Regime for the 2000–2010 Period and Fire Suppression

We then used available fire statistics for the period between 2000 and 2010 to examine the potential role of fire suppression processes in the study area. This period starts with the implementation in Catalonia of logical analyses of fire behaviour [29]. We therefore expect to find lower observed values of both fire regime descriptors during this period, in comparison to what would have been the case under the baseline scenario of no changes in firefighting strategy. In other words, we expect that considering fire suppression in the model will lead to a better match with the observed fire regime for the 2000–2010 period.

We defined nine fire suppression treatments (Table 1): the first one did not involve any fire suppression strategy, whereas the remaining treatments involved active fire suppression, opportunistic fire suppression or a particular combination of both strategies. We considered three different levels of opportunistic fire suppression, according to the TSF value after which opportunities for fire suppression disappear due to shrub encroachment and forest regeneration (5, 10 and 15 years). Likewise, we used three distinct fire spread values as thresholds to define levels of active fire suppression. These three values correspond to prototypical situations of increasing difficulty for fire extinction: 30 (i.e. weak active fire suppression, including the extinction of fires that burn agriculture covers or back fire fronts in sclerophyllous forests), 70 (i.e. medium active suppression, including the extinction of fires burning sclerophyllous forests in flat conditions), and 95 (i.e. strong active suppression, including the extinction of backing fires, or fires descending fronts in pine forests). We ran 100 simulations replicates of the MEDFIRE model under each fire suppression treatment. We used meteorological records of summer weather conditions (including years such as 2003 with record summer temperatures [50]) to determine adverse (5 years) and non-adverse years (6 years) in the model. Thus, we simulated the fire regime obtained under different fire suppression strategies while accounting for observed climatic constraints.

**Table 1:** Definition of the nine fire suppression treatments as combinations of firefighting strategies.

		Fire suppression treatment (threshold values in parentheses)	
Treatment	Description	Active	Opportunistic
0	Base (no fire suppression strategy)	No	No
i	Weak opportunistic suppression	No	Yes (5 years)
ii	Medium opportunistic suppression	No	Yes (10 years)
iii	Strong opportunistic suppression	No	Yes (15 years)
iv	Weak active suppression	Yes (30)	No
v	Medium active suppression	Yes (70)	No
vi	Strong active suppression	Yes (95)	No
vii	Weak active & strong opportunistic suppression	Yes (30)	Yes (15 years)
viii	Medium active & strong opportunistic suppression	Yes (70)	Yes (15 years)

Threshold values were used to simulate different levels of suppression effectiveness. In the case of active suppression, the fire in a given cell was extinguished if the spread rate (a value between 0 and 100) was lower than the specified threshold value. In the case of opportunistic suppression, the fire was extinguished if the value of time since last fire (in years) was lower than the specified threshold.

If firefighting has impacted fire regime descriptors for the 2000–2010 period, we expect a lower overall burnt area and a smaller percentage of area burnt in large fires in the observed fire regime compared to the fire regime of simulations under the baseline scenario without firefighting. To test this hypothesis, we calculated, under the baseline scenario, the proportion of simulations replicates that produced fire descriptor values smaller than the observed values. These probabilities were calculated for the whole study area as well as for bioclimatic sub-regions. Because we controlled for climatic effects in our simulations, a low proportion of replicates meeting these conditions would indicate that fire suppression was indeed impacting the fire regime. In order to determine which firefighting strategies would be compatible with observed fire regime, we followed the same procedure as in the validation test. A given fire suppression treatment was deemed compatible with the observed fire regime if the observed values of both descriptors were within the 95% of simulated values. Given the spatial variation in fire regimes known to exist in Catalonia [35], we also compatibility was evaluated for both the whole region and the three bioclimatic sub-regions. While overall fire suppression efforts were similar during the study period in the different regions, we predicted active suppression to be less likely to lead to changes in fire regimes in sub-regions with increasing importance of wind driven fires [49] that have overall faster spread rates (ie. NE region, Figure 1).

### Effects of Fire Suppression on Fire Regime in a Context of Climate Change

In a final simulation exercise, we used the MEDFIRE model to evaluate the potential role of firefighting practices in constraining the regional fire regime at a medium time horizon (20 years) under different assumptions of climate change. We defined fire regime scenarios by generating combinations of climatic and suppression treatments. Climatic treatments were defined by specifying whether the percentage of adverse years is equal to the percentage observed for the pre-2000 period (C0∶35% adverse years); or it is 25% higher (C1∶60% adverse years); or 50% higher (C2∶85% adverse years), according to the trends described in [30], [47].

Given the potential strong, but still uncertain effect of fire suppression strategies, we decided to use a relatively wide range of fire suppression treatments in conjunction with climate scenarios. However, and after preliminary results that showed similar outcomes compared to the baseline scenario, we discarded weak fire suppression treatments (i, ii and iv). We ran 100 twenty-year simulations under each fire regime scenario and used two-way ANOVA to analyze the main effects and interaction between climatic and fire suppression treatments on the two descriptors of fire regime.

## Results

### Validation of the MEDFIRE Model

**Figure 4:**
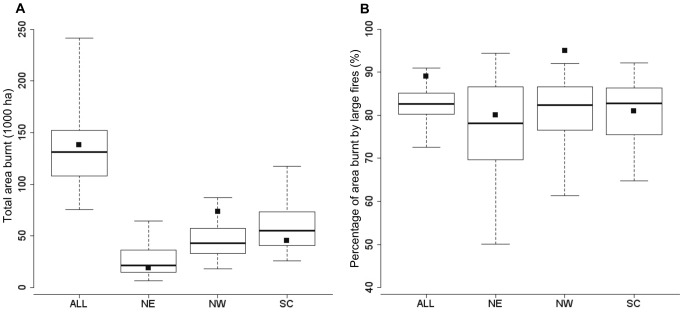
Statistical distributions for the total area burnt (A) and the percentage of area burnt by large fires (B) obtained after 100 simulations of the MEDFIRE model for the 1989–1999 period. Results are presented for the whole study area (ALL) and for the three bioclimatic sub-regions: North-East (NE), North-West (NW:) and South-Central (SC). Black squared dots indicate the observed values of total area burnt (A) and the percentage of area burnt by large fires (B) as reported in official statistics for this period. For all boxplots, lower and upper whiskers encompass the 95% interval, lower and upper hinges indicate the first and third quartile and the central black line indicates the median value.

Observed total area burnt and percentage of area burnt by large fires in the period 1989–1999 were compatible with fire regime distributions modelled by the MEDFIRE model calibrated with data from an earlier period (Figure 4A and B). When we analyzed fire regime descriptors at a regional level, we only obtained significant discrepancies for the percentage of area burnt by large fires in the NW region (Figure 4B). In the NW region, contrary to expectations of active fire suppression having an effect on fire regimes, the observed data showed more area burned than modelled by the MEDFIRE model. However, when we deleted from the data the single largest fire during the period, which occurred in 1994 and accounted for 28% of the total burnt area in the whole period, the observed data for this region was also within the 95% confidence intervals delivered by the calibrated model (Figure S1).

### Reproducibility of the Fire Regime for the 2000–2010 Period and Fire Suppression

**Figure 5:**
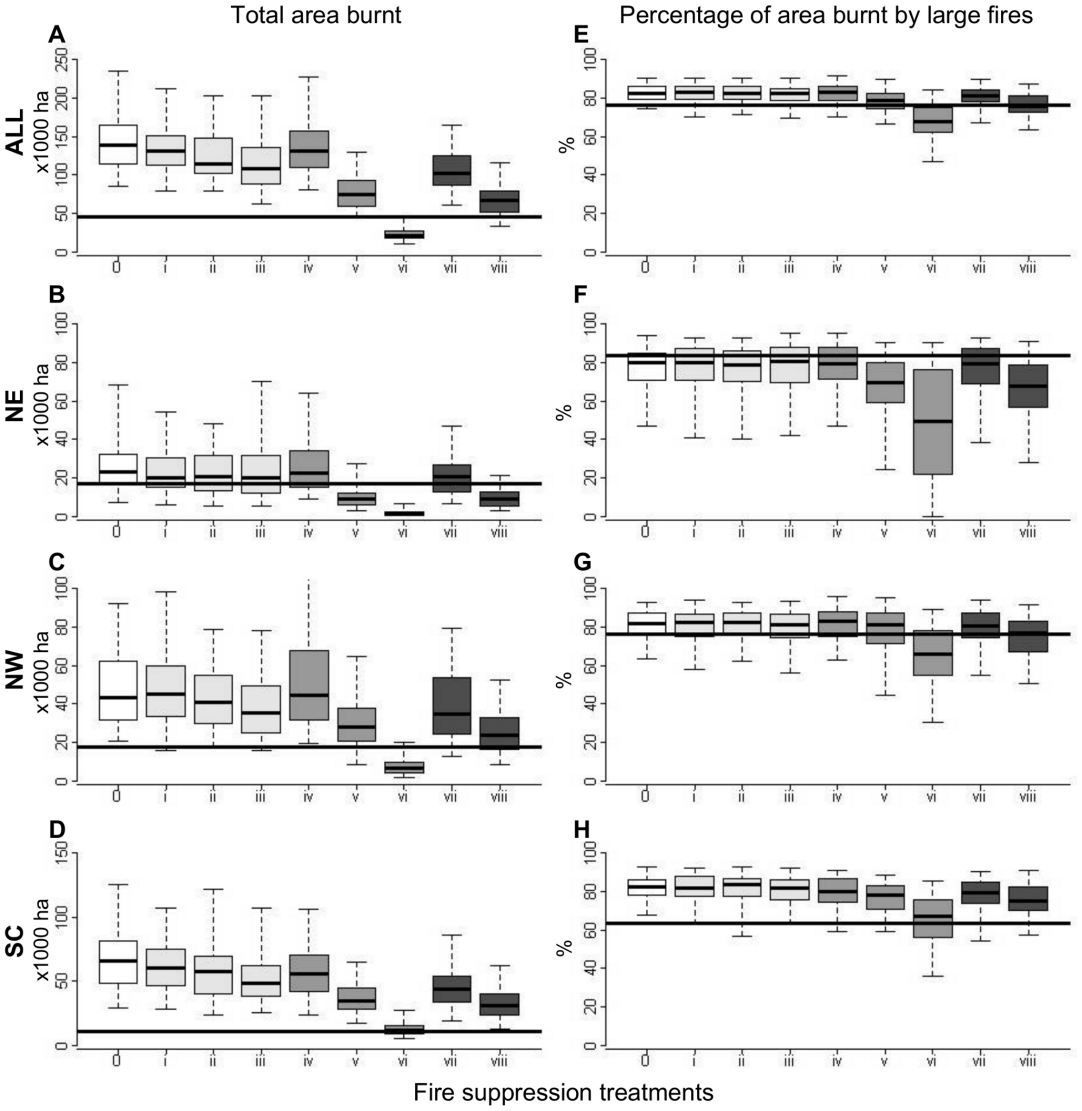
Statistical distributions for the total area burnt (A, B, C, D) and the percentage of area burnt by large fires (E, F, G, H) obtained after 100 simulations of the MEDFIRE model for the 2000–2010 period. Results are presented for the whole study area (ALL: plots A and E) and for the three bioclimatic sub-regions: North-East (NE: plots B and F), North-West (NW: plots C and G) and South-Central (SC: plots D and H). Scenarios without suppression are represented in white box-plots, opportunistic suppression scenarios in light grey, active suppression scenarios in medium grey and combined suppression scenarios in dark grey. Black horizontal lines indicate the observed values of total area burnt (A to D) or the percentage of area burnt by large fires (E to H) as reported in official statistics. Lower and upper whiskers indicate the 5% and 95% quartiles, lower and upper hinges indicate the first and third quartile and the central black line indicates the median value.

When we inspected the outputs of the MEDFIRE model for the 2000–2010 period, we found considerable variation across fire suppression treatments in the total amount of area burnt; with average reductions over the no-suppression scenario of up to 84% (compare treatments 0 and vi in Figure 5A).

The amount of area burnt reported by official statistics during the 2000–2010 was around 43,000 ha. This value is very small compared to the values obtained in our simulations under the treatment without fire suppression (Figure 5A). Indeed, none of the simulation replicates under this scenario produced values of the total area burnt below the observed value. In turn, our model simulations without fire suppression tended to overestimate the observed percentage of area burnt by large fires (Figure 5E). When considering together the joint distribution of both descriptors, we obtained that the probability of having simulated values lower than observed for both variables was zero in the baseline scenario. Furthermore, the NE was the only sub-region where simulated regimes without fire suppression were compatible with observed statistics: 28% of simulations produced lower values of both fire regime variables in the NE sub-region, whereas the same percentage was 1% for NW and 0% for SC.

Only treatments including medium active fire suppression (treatments v and viii) were compatible with observed fire statistics for the study period in Catalonia (Figure 5 A). The observed total area burnt in the NE sub-region was incompatible only with the treatment involving strong active fire suppression (treatment vi, Figure 5B). In other words and as predicted by the higher proportion of wind driven fires in this region, fire suppression was not needed to appropriately reproduce the observed fire regime in this sub-region. In the NW sub-region, several fire suppression strategies produced fire regime values compatible with observed data, although treatments involving medium active fire suppression, either alone (treatment v) or in combination with opportunistic suppression efforts profiting from fires up to 15 years old (treatment viii) fitted better the observations (Figure 5C and G). In contrast, only the treatment including a strong active fire suppression matched the observed fire regime in the SC sub-region (treatment vi; Figure 5D and H). In this case, a combination of strong active fire suppression and any opportunistic suppression treatment leads to similar results to those obtained from strong fire suppression alone (results not shown).

### Effects of Fire Suppression on Fire Regime in a Context of Climate Change

**Figure 6:**
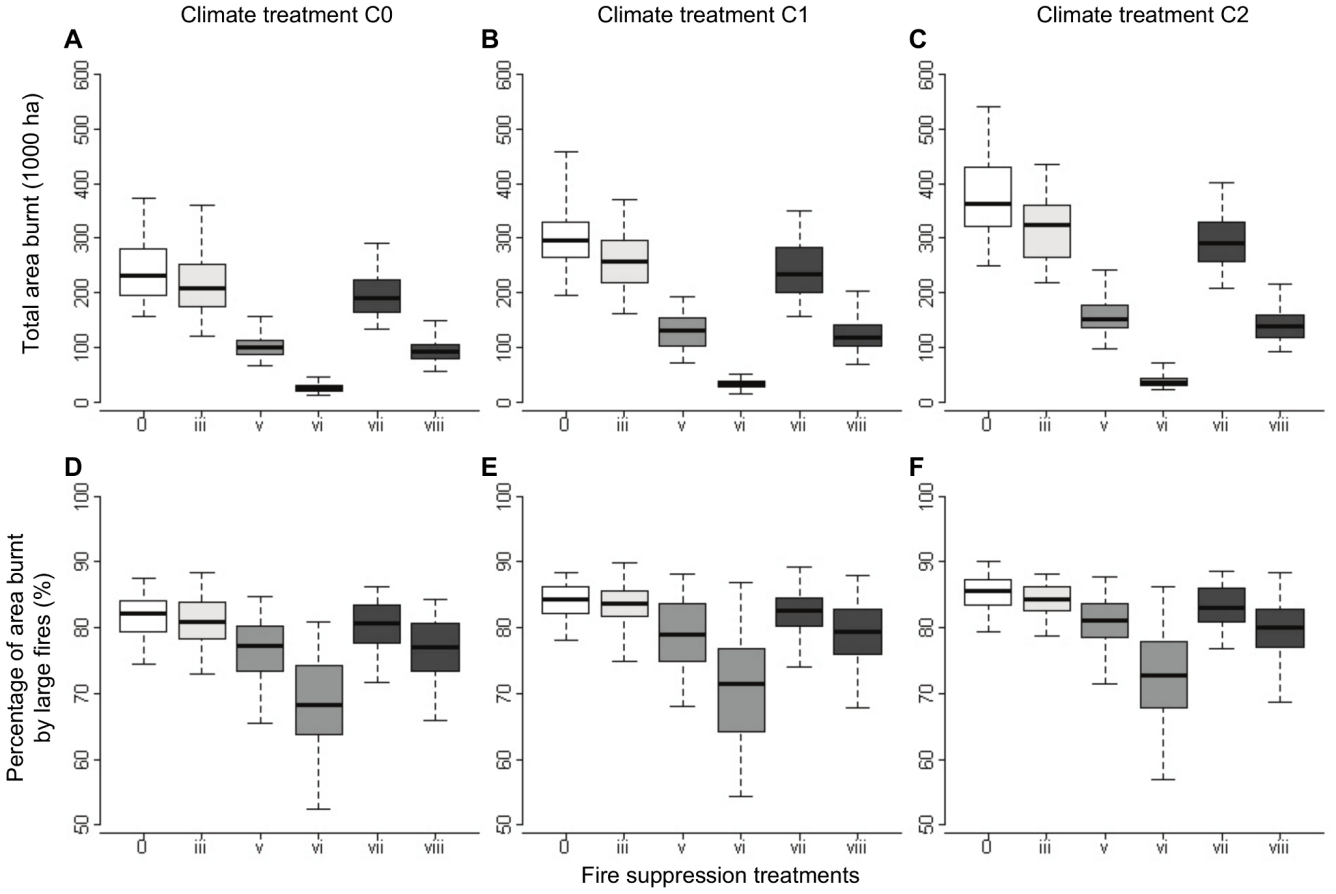
Statistical distributions for the total area burnt (A, B, C) and the percentage of area burnt by large fires (D, E, F), obtained after 100 twenty-year simulations of the MEDFIRE model under different fire regime scenarios. Scenarios were defined by combining climate treatments (C0, C1 and C2; defined in the main text) and fire suppression treatments (0, iii, vi, vii, viii; defined in Table 1). Lower and upper whiskers indicate the 5% and 95% quartiles, lower and upper hinges indicate the first and third quartile and the central black line indicates the median value.

The total amount of area burnt was highly dependent on climatic treatments as tested using ANOVAs (Figure 6; Tables S1). Scenarios involving an increase in the proportion of adverse climate years (C1 and C2) and no fire suppression (treatment 0) resulted in strong increases of up to 64% in the total area burnt compared to the corresponding scenario without climate change (C0). Fire suppression had also a strong effect on the total amount of area burnt. However, the degree to which fire suppression compensated for the effects of climate change depended on the firefighting strategy. Strong opportunistic fire suppression (treatment iii) had a relatively weak effect on the total area burnt in the baseline climatic scenario (C0), reducing total area burnt by about 9%. This percentage of reduction increased slightly to 11% under climate change scenarios C1 and C2 (Figures 6A–C). Active fire suppression had a much larger potential than opportunistic fire suppression to contribute to reductions in total area burnt. We obtained up to about 89% reduction in area burned in scenarios with strong active suppression (treatment vi), with little variation across climatic treatments. In addition, fire regimes obtained in these scenarios included shifts in fire size distributions. The area burnt by large fires decreased from about 82–85% in the absence of fire suppression to about 68–73% with strong active suppression (Figures 6D–F). When active and opportunistic suppression strategies were applied simultaneously, the burnt area reduction was slightly lower than the sum of the reductions obtained individually, indicating an overlap in the contribution of the two suppression strategies to reductions in area burnt. In particular, a weak active suppression combined with strong opportunistic suppression (scenario vii) involved about 9% reduction in area burnt compared with strong opportunistic suppression alone (scenario iii). The benefit of combining medium active suppression with strong opportunistic suppression (scenario viii), compared to medium active suppression alone (scenario v) was also about 9% in different climate change scenario (Figures 6A–C).

## Discussion

Large-scale changes in fire regimes are expected in a world in which climate change and human activities are on the rise. Recent reviews [4], [13] on the determinants of fire size distributions suggested that insights into the factors behind fire regimes would come from studies that combine empirical observations of past fires with the results of simulation models that use process-based mechanistic knowledge of fire occurrence and behaviour. Our model and methodology fitted this combined approach and allowed us to disentangle the relative effects of different determinants of fire regime in a Mediterranean region. In particular, we introduced the “opportunity” concept allowing us to constrain potential fire sizes by the use of opportunities related to either historical fire scars or to general firefighting efficiency. This allowed us to test and evaluate the impact of particular processes on shaping fire regimes. Our results support the view that active fire suppression is a key factor in determining short-term fire impacts. Furthermore, they also indicate that recent fire history has the potential to play a role in firefighting by offering suppression opportunities. Hence, we suggest that, if one aims at capturing critical drivers of fire regimes at the landscape scale, fire suppression should be explicitly integrated with climate change in the definition of future scenarios.

We also identified some potential avenues to implement this integration and address this question, albeit further examination of this complex issue is obviously needed. Assessments of fire suppression impacts on fire regimes are often performed using a long time perspective and do not include short-term changes in vegetation derived from the fire itself [51]. In the case of the complex dynamics of Mediterranean landscapes, fire regimes are likely to be the result of interactions involving vegetation, climate and human activities affecting fire ignitions, spread and suppression [8], [46]. While previous studies using fire models have shown that fire suppression was likely to have an impact on the fire regime [22], [52], very few studies have quantified with observational data the potential impact of fire suppression at the landscape scale and how this impact varies geographically. Using observed data for an eleven-year period (2000–2010), our results show that fire suppression efforts are overriding the expectations derived from adverse climate in determining the current fire regime in the short term in Catalonia. Other key parameters that determine changes in fire regimes, such as the number of ignitions, have not significantly decreased during the study period. Therefore, it is difficult to argue that they may be behind the low amount of area burnt observed. In two of the three regions analysed, the total amount of area burnt during recent years could not be explained without the inclusion of strong active suppression leading to reduced effective fire sizes. This is in line with our predictions following changes in firefighting policies in 1999 leading to more effective fire suppression. The likely exception to this rationale and supported by our analyses, is for sub-regions where wind driven fires with faster spread rates not linked to fuel characteristics [53] constrain the use of opportunities by fire fighters.

### Fire Suppression Strategies and Impact on Fire Regimes

Impacts of fire suppression on fire regimes are difficult to identify [54] and, in areas where this impact has been suggested, discussions arise regarding the degree to which reported temporal or spatial changes in fire regimes are in fact related to fire suppression efforts [55]. Previous studies based on model simulations suggested that fire suppression is likely to lead to either a larger number of large fires in the long run or to higher fire intensity [27], [52]. Moritz [10] showed that fire suppression has affected the characteristics of smaller fires to a larger extent than those of larger fires, supporting the claim that fire suppression could offset ecological risks posed by increasingly frequent human-caused fires in specific areas. Their findings contradicted the assertion that, in the absence of fire suppression, large fires would be constrained by more complex age-patch mosaics on the landscape. Moritz’s [10] conclusion that fire suppression does not cause large fires in the long term contradicts much of the current thinking behind ecosystem management in fire prone systems such as California’s shrublands [56]. Our results in Catalonia add some additional complexity to the study of this issue, because our study highlights the role of fire suppression as a major factor in this interplay leading to prevailing fire regimes, and identifies interactions between fire regime and fire suppression via landscape pattern, as a potentially important, albeit rarely considered mechanism driving fire regimes in humanised landscapes. For example, in our first simulation study, opportunistic fire suppression has the potential to contribute to the observed fire regime statistics involved, at least for some of the studied regions. This finding indicates that suppression efficiency is not independent of previous landscape history and the spatial autocorrelation of fire occurrence and that fire suppression may interact with recent fire scars to provide opportunities limiting the effective size of new fires. On the other hand, in our second simulation study, we found a large degree of overlap between the contribution of medium active suppression and strong opportunistic suppression to the reduction of total burnt area. In other words, effective fire suppression may lead to a reduction in the availability of locations for future opportunistic suppression. This casts some doubt on the claim that effective fire suppression may be associated to a larger area burnt in the long run because of fuel accumulation (fire paradox). Rather, the final outcome will be dependent on the relative contributions of opportunistic and active fire suppression strategies through time and their interactions with climate variability and landscape patterns. At present, opportunistic fire suppression can only explain a relatively small percentage of reductions in effective fire size in our scenarios and cannot explain *per se* recent, short term, fire size distribution patterns in our study region. This suggests that the current high fire suppression efficiency may be leading to changes in the relative weight of factors in the future fire regime by decreasing the opportunities that arise from past fires.

### Fire Suppression and Mitigation of Climate Change Effects on Fire Regimes

Adverse climatic conditions are a key element in determining current fire regimes in Mediterranean areas [4], [12]. Our scenarios including increases in the proportion of climatically adverse years, indicated that the total area burnt may considerably increase in the forthcoming years if the proportion of climatically adverse years rise in line with increases in summer temperatures observed during the last decennium of the XXth century [30]. Furthermore, our results support the view that very high levels of fire suppression would be needed if these figures are to be reduced or compensated [34], [51]. Our results indicate that opportunistic fire suppression alone has a limited capacity to compensate for increased impacts of climate change on the fire regime. While climate change increases the amount of burnt area and thus the number of opportunities for effective fire suppression, suppression itself reduces future opportunities. Given that climate and fire suppression exert opposite influences on fire regime by favouring or restricting the presence of large fires, the final outcome is likely to be that of an unstable equilibrium [49], [57]. Any large-scale compensation of climate change impacts on the fire regime will require largescale active fire suppression to be effective. While our results indicate that fire suppression efforts compatible with recent fire regime (including medium active suppression) may counteract the effects of climate change in the medium term (20 years), we did not include variability in fire suppression efficiency within scenarios. Firefighting techniques may be overwhelmed by simultaneity of fires or fires affecting heavily inhabited areas [8], [30]. Also, fires under future climate conditions could be more aggressive than current fires, thus pressing firefighting systems beyond their current extinction capacity [51]. Assessing the effect of variability in suppression efficiency linked to critical weather periods or fire types (e.g. crown vs. surface fires) [58] would allow gaining insight into the interactions determining current short- to medium-term fire regimes [35], [59].

### Avenues for Future Research

Further investigation of interacting mechanisms and feedbacks between factors potentially affecting fire impact is merited. Our results suggest that fire regimes in areas under strong human and climate change influence are not likely to be under stable fire regimes but rather show short term impacts of the idiosyncratic contributions of the different factors in the system. In the case of Mediterranean landscapes, one could predict a strong increase of fire impacts in the near future. However, it is not clear whether this will be the case under current levels of fire suppression and landscape changes leading to shifts in dominant vegetation and the expected feedbacks between these factors [8], [57]. Shifts from pine-dominated to oak-dominated stands [4], [12] may have an important role in further reducing overall landscape fire risk by offering enhanced firefighting opportunities. Modelling additional mechanistic (bottom-up) aspects of fire spread and fire extinction would be needed to determine whether the change in dominant tree species affects the fire regime in the long term in Mediterranean forests.

### Lessons Learned for Management

The concept of fire suppression opportunity associated with known and predictable factors affecting fire spread opens the way to landscape management approaches aiming at effectively changing fire regimes. In general, as the global emphasis on fire suppression policies increases, there will be a need to evaluate the effects of direct fire suppression and indirect fire management, through fuel modifications, on fire size distributions. There will also be a need to understand how fire regimes will be naturally affected by climatic changes operating through changes in fire weather conditions or changes in dominant forest cover types. Finally, the potential to alter fire regimes will involve the creation of new landscape configurations that may have critical impacts on biodiversity patterns. In the case of opportunistic fire suppression, interactions between past fire history and fire impact may increase the degree of autocorrelation in the spatial pattern of fire patches, potentially affecting ecological processes such as regeneration or colonisation of early successional species associated with fire [60].

## Supporting Information

Figure S1 **Statistical distributions for the total area burnt (A) and the percentage of area burnt by large fires (B) obtained after 100 simulations of the MEDFIRE model for the 1989–1999 period.** Results are presented for the whole study area (ALL) and for the three bioclimatic sub-regions: North-East (NE), North-West (NW:) and South-Central (SC). Black squared dots indicate the statistics of total area burnt (A) and the percentage of area burnt by large fires (B) omitting from the observed data the single largest fire occurred in 1994. Boxplot description is as in the manuscript.
(TIF)

Table S1 **Three-way ANOVA tables summarizing the results of twenty-year simulated fire regimes scenarios in terms of the total area burnt (A) and percentage of area burnt by large fires.**
(DOC)

Appendix S1 **Sub-model Description.** This appendix describes details of the two sub-models of the MEDFIRE model, the fire sub-model Forest growth and the vegetation dynamics sub-model.
(DOC)

Appendix S2 **Initialization of model variables.** This appendix describes the main data sources employed to initialize spatial state variables and parameters used in the MEDFIRE model and described in Appendix S1.
(DOC)
