# Supplementary figures and images for: How Fire History, Fire Suppression Practices and Climate Change Affect Wildfire Regimes in Mediterranean Landscapes
Source: PLoS One. 2013 May 2;8(5):e62392. doi: 10.1371/journal.pone.0062392 (PMC3642200; doi:10.1371/journal.pone.0062392)

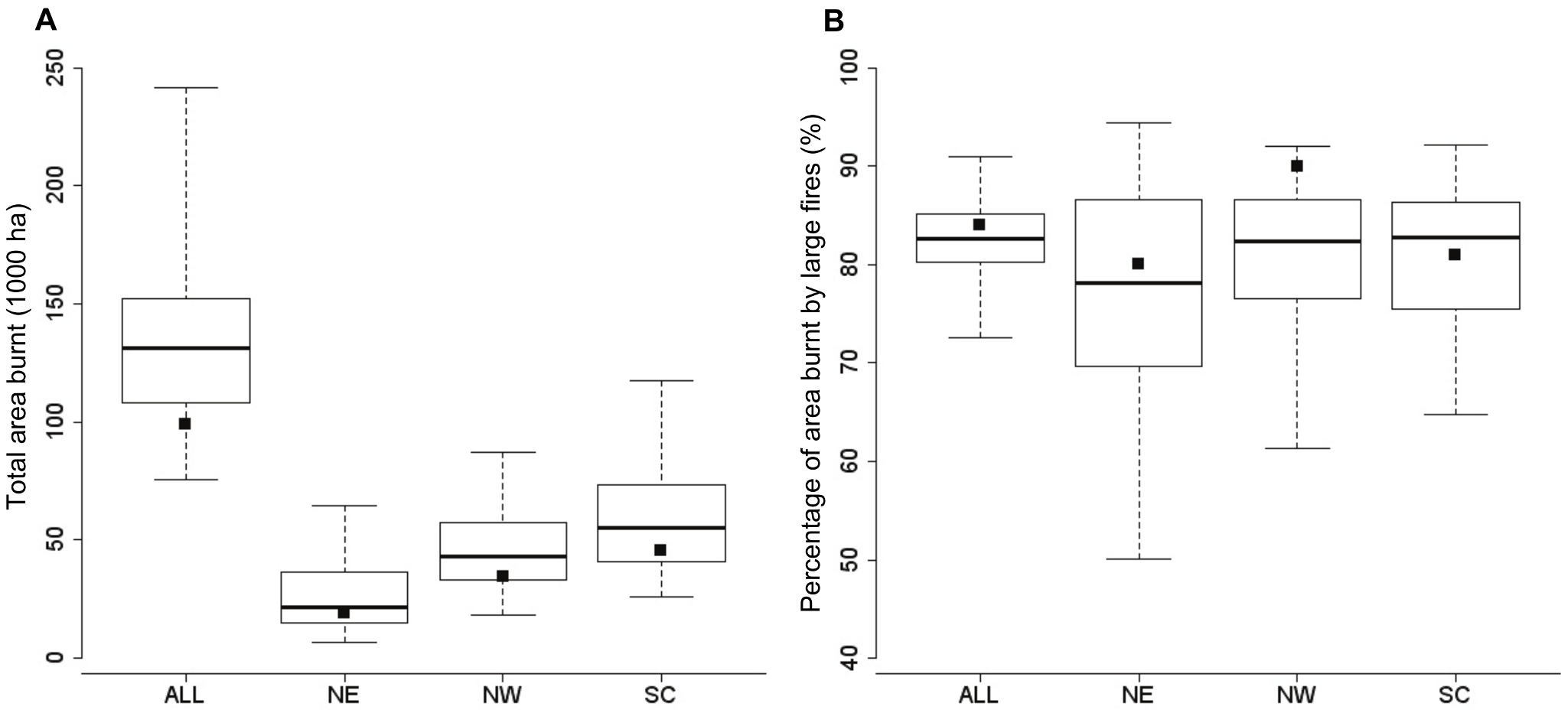

Supplement: Figure S1 — Statistical distributions for the total area burnt (A) and the percentage of area burnt by large fires (B) obtained after 100 simulations of the MEDFIRE model for the 1989–1999 period. Results are presented for the whole study area (ALL) and for the three bioclimatic sub-regions: North-East (NE), North-West (NW:) and South-Central (SC). Black squared dots indicate the statistics of total area burnt (A) and the percentage of area burnt by large fires (B) omitting from the observed data the single largest fire occurred in 1994. Boxplot description is as in the manuscript. (TIF) [file pone.0062392.s001.tif]
