# Supplementary material for: How Fire History, Fire Suppression Practices and Climate Change Affect Wildfire Regimes in Mediterranean Landscapes
Source: PLoS One. 2013 May 2;8(5):e62392. doi: 10.1371/journal.pone.0062392 (PMC3642200; doi:10.1371/journal.pone.0062392)
Supplement: Table S1 — Three-way ANOVA tables summarizing the results of twenty-year simulated fire regimes scenarios in terms of the total area burnt (A) and percentage of area burnt by large fires. (DOC) [file pone.0062392.s002.doc]

**For online publication only**

**Table S1.** Three-way ANOVA tables summarizing the results of twenty-year simulated fire regimes scenarios in terms of the total area burnt (A) and percentage of area burnt by large fires (B).

A) Total area burnt (adjusted R^2^ = 0.8313)

Factor Df Sum Sq Mean Sq F value Pr(>F)

Climate 2 1655066 827533 394.796 <0.0001

Fire Supp. 5 16488218 3297644 1573.224 <0.0001

Climate:Fire Supp. 10 476973 47697 22.755 <0.0001

Residuals 1782 3735260 2096

B) Percentage of area burnt by large fires (adjusted R^2^ = 0.4581).

Factor Df Sum Sq Mean Sq F value Pr(>F)

Climate 2 3791 1895.5 72.6430 <0.0001

Fire Supp. 5 36252 7250.4 277.8671 <0.0001

Climate:Fire Supp. 10 89 8.9 0.3402 0.9702

Residuals 1782 46498 26.1
