# Supplementary material for: How Fire History, Fire Suppression Practices and Climate Change Affect Wildfire Regimes in Mediterranean Landscapes
Source: PLoS One. 2013 May 2;8(5):e62392. doi: 10.1371/journal.pone.0062392 (PMC3642200; doi:10.1371/journal.pone.0062392)
Supplement: Appendix S1 — Sub-model Description. This appendix describes details of the two sub-models of the MEDFIRE model, the fire sub-model Forest growth and the vegetation dynamics sub-model. (DOC) [file pone.0062392.s003.doc]

**For online publication only**

**APPENDIX S1: Sub-model Description**

This appendix describes details of the two sub-models of the MEDFIRE model, the fire sub-model Forest growth and the vegetation dynamics sub-model.

*Fire sub-model*

The fire sub-model is responsible for simulating the impact of a given fire regime in the landscape of a given area. This landscape event is scheduled once every year in summer. The sub-model begins by determining either from a preselected distribution or an input table whether the current summer is climatically wet (normal) or dry (adverse). Then, a total annual extent to be burnt is drawn from a statistical distribution, which differs depending on whether the summer is climatically normal or adverse (*AnnualBurnDistNorm* and *AnnualBurnDistSevr*). For each fire, the sub-model stochastically selects the target fire size, an ignition point and the fire-spread type (either relief- or wind-driven). As for the total annual extent to be burnt, distinct fire size distributions are used for climatically normal and adverse years (*FireSizeDistNorm* and *FireSizeDistSevr*). If fire suppression is not active, the fire is allowed to spread from its ignition point until the burnt extent equals the target fire size. In contrast, if fire suppression is active not all the cells potentially affected by a fire will be effectively burnt. Ignitions are generated and fires spread one after the other sequentially until the total annual extent to be burnt is reached. All burnable land cover types (see Table 1 in Appendix 3), effectively or non-effectively burnt, are counted when calculating total burnt extents.

1. *Ignition point*: Ignition points are restricted to occur in cells with burnable land cover type (*LCT*) (i.e. urban, water and rock covers are excluded). Ignition points are stochastically determined by randomly picking a grid cell with weights given by *W_Ignition_* [1]. This value is derived from an input layer that describes a basic probability of ignition for each grid cell (*ProbIgnition*),
2. *Fire spread and burning*: Before starting a new fire, a value for its fire size is drawn from a statistical fire size distribution. This value sets the target extent to be burnt. The process of fire spread is as follows based on [2]. A given cell that is set to burn is called an *active cell*. The first active cell is the grid cell selected as ignition point. For each active cell, its eight immediate neighbors are considered as cells where the fire can spread. We refer to these as *spreading cells*. For every spreading cell, the model calculates a spread rate (*SR >* 0), which is a dimensionless number but is used to determine the order in which spreading cells are processed (removed from the event queue). Cells with low *SR* values are processed later than those with high *SR* values. When a given spreading cell is processed, the model then calculates the probability of burning (*P_burning_*) and determines whether the cell burns or not using a Bernoulli trial. If the cell burns it becomes an active cell, and the spreading algorithm is processed for that cell. Otherwise the fire front at this point is stopped. The spatial pattern of a given fire arises as a result of differences among cells in the rate of spread and probability of burning. In other words, different combinations of spread rate and propensity to burn control fire shape and the proportion of unburned islands. Fires generally burn until their burnt extent equals the pre-specified fire size. Hence, the fire extent may be reached before slow-spreading cells are processed. Once the target extent is reached, all active cells on the front are stopped and the fire is completed. To ensure that fires do no stop before the target extent is reached, cells on the front that do not burn are kept in a randomly sorted list and the fire front is re-ignited if needed from these cells. Otherwise, these remain unburned.

Two basic fire spread patterns are considered: relief-driven base versus wind-driven base spreads [3]. The fire spread type layer (*SpreadType*) contains the proportion of topographic fires for each cell in the grid. A Bernoulli trial for the ignition location with the probability of relief-driven fires taken from this proportion is used to determine the specific spread type for each simulated fire. To model the influence of fuel in the rate of spread, a *Base* value is modified according to a *Fuel* multiplier as a proxy of fuel load and aspect of the *spreading cell* following the spread rate formulation in [4,5]. The *Base* and *Fuel* values are combined to calculate the rate of spread (*SR*) as:

$SR = 1-e^{-(Base\cdot Fuel)}$ (A1)

being higher for uphill fronts, wind-aligned fronts, southern slopes and higher for specific *LCT* covers and fuel loads [2,6-8]. Whether a cell burns or not mainly depends on the spread rate. Slower fires are assumed to be more likely to go out due to local conditions (slow fires are most likely lower intensity, such as heading downhill, against the wind, across lower flammable types, etc.). The probability of burning is simply calculated as:

$P_{burning} = SR^{SR\_BurnExp}$ (A2)

where exponent *SR_BurnExp* (≥ 0) controls the relationship between spread rate and probability of burning.

- 1. *Relief-driven Base spread*: The basic spread rate in a relief-driven fire is modeled as follows: First, the difference in altitude between the spreading cell and the active cell is assessed using *Elevation* spatial variable. This difference allows calculating the slope of the burning front (*estFireSlope*), which is afterwards bounded between –0.5 and +0.5 (±50%). The slope of the burning front is used to calculate a base rate of spread following:

*Base* = (1+*rSlope*) (*^estFireSlope^*^+0.5)^ (A3)

where *rSlope* (≥ 0) specifies the extent to which slope modulates the spread rate. Note that *Base* = 1 when the slope of the burning front is -0.5 (downhill) and *Base* > 1 for higher values of slope.

- 1. *Wind-driven Base spread*: The basic spread rate for a wind-driven fire is calculated taken into account the local wind direction since it has significant effect on fire spread [9]. First, *fire spread direction* is defined as the vector from a *fire anchor point* to the spreading cell. The model then measures the angle in degrees (*degreesOffWind*) between the *fire spread direction* and the main wind direction (given at cell level by *Wind* spatial variable). This value, which ranges from 0 to 180 degrees, is used to calculate the basic spread rate following the function:

*Base* = (1+*rWind*) ^((180 –^ *^degreesOffWind^*^) /180)^ (A4)

where *rWind* (≥ 0) specifies the extent to which *degreesOffWind* modulates the spread rate. Using this formula, *Base* = 1 when the fire is spreading against the wind (*degreesOffWind= 180*) whereas *Base* > 1 for fire fronts with lower angles between the main wind direction and the direction of fire spread. The initial fire anchor point for any fire is the ignition point. As fire spreads, however, if the local fire spread direction deviates significantly (> 45 degrees) from the direction from the anchor point (e.g. due to barriers such as urban or rock, or due to slower spread or local fire extinctions), the anchor point is updated. In this way broad scale fire direction can reduce bias from spreading within a grid (to eight neighbours on 45 degree angles), yet local effects that influence direction can be included to ensure fire direction responds to landscape structure. The fire anchor points tends to be located in areas that cause discontinuous changes in fire direction, such as non-burnable LCTs, areas with slower spread or low probabilities of burning, areas with local fire suppression, etc). After passing such areas, fire spread rate increases again in the direction of the wind.

- 1. *Fuel load spread*: Fuel load (*Fuel*) is calculated using the *LCT*, *TSF* and *Aspect* values of the *spreading cell*. *TSF* values are re-scaled to the [0-1] interval and the result is stored as *TSFExp*. Aspect is used to modulate fuel load by setting *AspExp* = -1 when the spreading cell is oriented to North, *AspExp* = +1 when it is oriented to South and *AspExp* = 0 otherwise. Each burnable *LCT* has its corresponding *flammability* quality parameter, *f_LCT_* (≥ 1). The fuel load is then calculated as:

$Fuel = (1+rAspect)^{AspExp}\cdot(1+rTSF)^{TSFExp}\cdot(rLCT \cdot f_{LCT})$ (A5)

where *rAspect*, *rFuel* (both ≥ 0) and *rLCT* (> 0) specify the extent to which differences in *Aspect*, *TSF* and *LCT flammability*, respectively, modulate the fuel load.

1. *Fire suppression*: Two distinct fire suppression strategies are implemented, both related to the concept of fire fighting opportunity. The active fire suppression strategy concerns opportunities generated in areas where *SR* is lower than a pre-specified threshold (*SRthreshFF*). The passive fire suppression strategy is related to opportunities given by low fuel loads and consists in suppressing the fire whenever *TSF* is lower than a pre-specified threshold (*TSFtreshFF*). For any of the two fire suppression strategies, if a cell is said to burn but complies with the required condition it will not burn. However, the model allows the fire to continue spreading from that cell (but without effectively burning), so that areas that would have been reached via spread beyond the suppression opportunity point also do not burn.
2. *Fire effects*: Cells that are effectively burned have *TSF* set to 0. The fire extent is calculated as the number of cells that effectively burned plus the number of cells that would have burned but did not because of fire suppression of all burnable *LCT* cells reached via spread.

*Vegetation dynamics sub-model*

The vegetation dynamics sub-model is responsible for updating the *LCT* state variable. This landscape event is scheduled once every year at the end of the year. The two ecological processes implemented in this sub-model are: (1) vegetation regeneration following fire disturbances (i.e., post-fire vegetation transitions); (2) natural succession from shrubland to forest (only changes between shrubland and forest classes are allowed and other natural covers are considered stable).

1. *Post-fire vegetation regeneration:* Post-fire changes in *LCT* represent the outcome of vegetation regeneration dynamics after the impact of fire in a given area. Only those cells that have burnt in the current year are allowed to undergo a *LCT* change. Post-fire transitions in dominant species are implemented according two approaches: non-spatial stochastic transitions or neighborhood species contagion. This is implemented by allowing the updated *LCT* value of some target cells to be based on the current *LCT* values of their neighbors. Otherwise, a non-direct, deterministic regeneration approach is applied defining post-fire changes as transition probabilities [10] based on data from [11]. Whether neighbor contagion transitions happen is determined using a Bernoulli distribution with success probability *pNeighbContag*.
   1. *Spatially autocorrelated transitions*: When neighbor contagion is used, any neighbor (at 150 m radius) of the target cell available is considered appropriate for contagion if: (1) were also burnt this year; (2) have the same pre-fire *LCT* and *TSF* values; and (3) their *LCT* value has been already updated. The updated *LCT* value is copied from a neighbor randomly chosen among those that are appropriate for contagion. If none of the neighbors is considered appropriate for contagion then the transition is not spatially autocorrelated.
   2. *Non-spatially autocorrelated transitions*: Post-fire *LCT* transition probabilities (*postFireSucc*) depend on the pre-fire *LCT* value and are regionalized following the bioclimatic regions (*BioRegion* spatial variable). Given that transitions after fire are also known to depend on time since the previous last fire [12], we applied a different transition probability table for recent re-burnt areas (*postFireSuccReburnt*) when less than *CanopySeedAge* years have passed between the last fire event and the current one. Vegetation regeneration patterns are strongly linked to aspect value [13]. Accordingly, we increased the amount of transitions to shrubland in southern slopes using an aspect factor (*SppAspectFactor*).
2. *Succession from shrubland to forest*: Each year, shrubland cells are allowed to become forests. The probability of becoming forested (*P_shrub2forest_*) is calculated as a logistic function:

$\mathrm{logit}(P_{shrub2forest}) = a+b\cdot TSF_{shrub}\cdot ForNeigh$ (A6)

where *ForNeigh* is product of the proportion of neighbors (150 m radius) that are considered adult forests (i.e. cells which *LCT* is a tree species and its *TSF* ≥ *MatureForest*), *TSF_shrub_* represents the age of the shrubland itself, and *a* and *b* are, respectively, the intercept and slope of the linear predictor. In the case that shrubland becomes a forest, the dominant tree species is chosen using a multinomial distribution where the probabilities are calculated using the number of adult forests of each *LCT* among the neighbors (150 m radius) of the target cell. The number of neighbors of each forest type is weighted by a seed pressure factor (*SeedPressure*) since not all tree species have the same colonization capability of new areas in a Mediterranean landscape [14].

**References**

1. Bar Massada A, Syphard AD, Hawbaker TJ, Stewart SI, Radeloff VC (2011) Effects of ignition location models on the burn patterns of simulated wildfires. Environ Model Softw 26: 583–592. doi:10.1016/j.envsoft.2010.11.016.

2. He HS, Mladenoff DJ (1999) Spatially explicit and stochastic simulation of forest-landscape fire disturbance and succession. Ecology 80: 81–99.

3. Rothermel R (1972) A mathematical model for predicting fire spread in wildland fuels. USDA Forest Service Research Paper INT USA.

4. He H, Li W, Sturtevant B, Yang J, Bo S, et al. (2005) LANDIS 4.0 users guide. LANDIS: a spatially explicit model of forest landscape disturbance, management, and succession. U.S. Department of Agriculture, Forest Service, North Central Research Station. p.

5. Sturtevant B, Miranda B, Scheller R (2010) LANDIS-II Dynamic Fire System Extension (v1. 0) User Guide. landis-ii.org. pp.

6. Millington JDA, Wainwright J, Perry GLW, Romero-Calcerrada R, Malamud BD (2009) Modelling Mediterranean landscape succession-disturbance dynamics: A landscape fire-succession model. Environ Model Softw 24: 1196–1208. doi:10.1016/j.envsoft.2009.03.013.

7. Seidl R, Fernandes PM, Fonseca TF, Gillet F, Jönsson AM, et al. (2010) Modelling natural disturbances in forest ecosystems: a review. Ecol Model: 1–22. doi:10.1016/j.ecolmodel.2010.09.040.

8. Piñol J, Beven K, Viegas D (2005) Modelling the effect of fire-exclusion and prescribed fire on wildfire size in Mediterranean ecosystems. Ecol Model 183: 397–409. doi:10.1016/j.ecolmodel.2004.09.001.

9. Sharples JJ, McRae RHD, Weber RO (2010) Wind characteristics over complex terrain with implications for bushfire risk management. Environ Model Softw 25: 1099–1120. doi:10.1016/j.envsoft.2010.03.016.

10. Balzter H (2000) Markov chain models for vegetation dynamics. Ecol Model 126: 139–154. doi:10.1016/S0304-3800(00)00262-3.

11. Rodrigo A, Retana J, Pico FX (2004) Direct regeneration is not the only response of Mediterranean forests to large fires. Ecology 85: 716–729.

12. Díaz-Delgado R, Lloret F, Pons X, Terradas J (2002) Satellite evidence of decreasing resilience in Mediterranean plant communities after recurrent wildfires. Ecology 83: 2293–2303.

13. Pausas JG, Carbó E, Neus Caturla R, Gil JM, Vallejo R (1999) Post-fire regeneration patterns in the eastern Iberian Peninsula. Acta Oecologica 20: 499–508. doi:10.1016/S1146-609X(00)86617-5.

14. Verdú M (2000) Ecological and evolutionary differences between Mediterranean seeders and resprouters. Journal of Vegetation Science 11: 265–268.
