# Supplementary material for: How Fire History, Fire Suppression Practices and Climate Change Affect Wildfire Regimes in Mediterranean Landscapes
Source: PLoS One. 2013 May 2;8(5):e62392. doi: 10.1371/journal.pone.0062392 (PMC3642200; doi:10.1371/journal.pone.0062392)

**For online publication only**

**Appendix S2: Initialization of model variables**

This appendix describes the main data sources employed to initialize spatial state variables and parameters used in the MEDFIRE model and described in Appendix 1.

**1 – Spatial state variables**

*Land cover type (LCT)*

*LCT* describes land cover types and dominant tree species in forest areas of the region in the year 1989 (validation test) and year 2000 (Table 1). Information of non-forest categories was obtained from the second and third Land Cover Maps of Catalonia (LCMC) describing land cover from aerial photos [1]. Dominant tree species per cell were extracted from the Spanish Forest Map [2,3] and National Forest Inventory (NFI) data available for Catalonia [4]. The spatial distribution of each dominant tree species or species grouping (Table 1) was derived from the third NFI inventory data by applying kriging interpolation techniques [5] allowing the generation of a continuous layer of information from data measured in NFI forest plots.

*Time since last fire*

The time since last fire (*TSF*) was derived from fire historical statistics and accounted for the number of years since the most recent wildfire at a site-specific cell level. The wildfire data used consist of fire perimeters larger than 50 ha that occurred in Catalonia between 1975 and 1999 mapped at 1:50.000 [6,7]. The data set included 319 fires with a total area of 295,554 ha. Non-burned areas were assigned a default value of 200 years. The initialization of *TSF* for the validation test was based on fire perimeters from the 1975-1988 period.

*Ignition probability*

Natural and human factors associated with complex landscape drivers influence the spatial pattern of fire ignitions. The likely occurrence of ignitions may be estimated using available regional information on observed ignitions in historical fires and environmental information. The Catalan regional government has developed a layer describing the probability of fire ignition in the region (basic fire risk map) from anthropogenic and biophysical environmental variables [8]. The explanatory variables considered to categorize the basic fire risk (from 1 to 10) were historical ignition records, composition and structure of the vegetation, topography and climatic factors related with fire frequency and fire intensity [9,10]. The probabilities assigned to the basic fire risk map categories were based on the frequency of fire occurrence between 1975 and 1988 and used as inputs for the MEDFIRE spatial variable *ProbIgnition*.

*Other spatial state variables*

Other landscape features were represented by static spatial variables with the same initial conditions for all simulation runs (Table 2).

*BioRegion:* We defined bioclimatic regions from [10] allowing to introduce regionalization in post-fire transition probabilities. *SpreadType* The fire spread type spatial variable was included to determine the percentage of predominant fire spread pattern type (wind driven versus topographic) in a region based on the homogeneous fire regime zoning defined by [11]. *Elevation* and *Aspect* variable values were computed from the 100 meter elevation model available for the region. *Wind* data from 194 meteorological stations (heterogeneously distributed along Catalan territory) for a 10 year period was provided by the regional climate center [12]. Dominant monthly wind direction data in summer (June to September) were processed to identify the prevailing wind direction in each station and fire regime zoning using spatial interpolation [12].

**2 – Initialization of fire sub-model parameters**

The fire sub-model requires as initial spatial variables and parameters: (1) distributions describing the fire regime, (2) the variables modulating ignition, fire spread and burning processes, and (3) preselected thresholds allowing fire suppression to be successful. Table 4 summarizes the initial values of all the sub-model variables.

*Fire regime descriptors*

Essential fire regime attributes in the MEDFIRE model were defined as the total area burnt per year and fire size distributions. These are specified as input distributions derived from the available fire historical statistics described above. Both distributions (annual area burnt and fire sizes) differed between relatively wet years (normal) and climatically adverse years characterized by a high number of dry periods with high fire risk derived from [13]. Available daily data in six meteorological stations for 1975-1988 period (provided by *Agencia Estatal de Meteorología*; http://www.aemet.es) was used to identify years with high maximum temperatures and low precipitation (as a proxy for adverse weather conditions leading to high fire risk). Calculation of the climatic severity for the 1975–1988 period allowed the classification of fires in two categories and served to fit the required input distributions. Annual area burnt distributions (*AnnualBurnDistNorm* and *AnnualBurnDistSevr*) follow a log normal distribution with parameters μ-mean and σ-standard distribution [14], while fire sizes distributions (*FireSizeDistNorm* and *FireSizeDistSevr*) are described as power law distributions [15]. The number of fires *N* with size greater than *S* exhibits a potential relationship with the fire size (N ≈ f(S^-β^)), thus we fitted a linear model (α-offset, β-slope) to the relationship between log(*N*) and log(*S*) (see Table 3).

*Calibration of fuel fire spread rate parameters*

The probability of fire spreading into any of the neighbors of a burning cell was calculated in MEDFIRE as a function of slope or wind (base spread types), aspect factor modified by land cover flammability quality (*f_LCT_*) and fuel load (using *TSF* as a proxy for fuel accumulation) [16]. We first calibrated basic functions determining the role of relief, wind and aspect (*rSlope*, *rWind,* *rAspect*) and the probability of burning (*SR_BurnExp*) from the literature [17-19] and initial preliminary examinations of the patterns reproduced by the model in different parameters combinations in relation to the spread rate patterns observed in real fires with homogeneous land cover characteristics (Table 3).

Flammability of different vegetation is more difficult to estimate, and the theoretical functions available for relief or wind spread behaviors are scarce for the Mediterranean region [20,21]. Therefore, we calibrated the fuel related and the land cover flammability parameters (*rTSF,*  *rLCT* and *f_LCT_*) of the base MEDFIRE model by searching for a behavioral combination [22] of flammability parameters able to reproduce landscape patterns generated by selected historical fire perimeters. We run the model with *LCT* and *TSF* state variables initialized in the year 1989, constraining the fire ignition occurrence to observed ignitions, and simulating the spread pattern of both spread types, wind- and relief-driven, according to the observed types of the fire selected in the subset. The calibration test consists in the comparison of the percentages of burnt land cover types per fire within the observed fire perimeters and within the simulated perimeters. To obtain flammability parameters, we searched for a combination of parameters for which the mean percentage of the area burnt per fire for each of the five selected *LCT*-clusters (*Pinus* and other conifers, *Quercus* species, other tree species, shrubland and cropland) was within the 95% confidence interval obtained from the modeled distribution (Figure 1). The *LCT* for this calibration exercise was generated from the Second National Forest Inventory (NFI2) and available land cover maps (LCMC first version, [1], and MCA, *Mapa de Cultivos y Aprovechamientos*, [23]) using the same approach explained above for *LCT* in year 2000.

**3 – Initialization of vegetation dynamics sub-model parameters**

The ecological processes implemented in this sub-model are aimed to describe vegetation regeneration following fire disturbance and natural succession from shrubland to forest. Table 6 summarizes the initial values of all the sub-model variables.

*Post-fire vegetation regeneration*

Variables dealing with vegetation dynamics include detailed post-fire regeneration parameters implemented as dominant tree species transition probabilities based on [24]. Post-fire transition probabilities (*postFireSucc*) were modified to deal only with mono-specific forests (Table 7) and according to three additional factors. (i) Aspect factor (ie. the probability of shrub postfire transitions (*SppAspectFactor*) was increased ten fold in southern slopes [25], (ii) Time since last fire affecting postfire regeneration of *Pinus halepensis*, the only clearly serotinous species [26] developing a large canopy seed bank by 15 years after fire disturbance in post-fire stands [27]. In recent re-burnt areas (*TSF* < *CanopySeedAge* = 15 years), the persistence probability of *Pinus halepensis* decreased and the posrt-fire transition of this species to shrubland increased to 70% (*postFireSuccReburnt* variable). (iii) Bioclimatic regions determining the kind of transitions possible within a given region. *Pinus pinea* and other conifers pre-fire stands did not change to *Quercus suber* but to *Quercus ilex* in SC region. In NW region, the same two *LCT* post-fire transition probabilities to *Quercus suber* decreased for proportional increases in the transition probabilities to *Quercus ilex* and Other *Quercus*. Conifers in NW become *Quercus suber* and *Quercus ilex* with the same proportion [24]. The weight of the spatial autocorrelation process in the post-fire transitions (*pNeighbContag*) was set to 0.40.

*Succession from shrubland to forest*

Natural succession defined as temporal changes in dominant species was only modeled for the shrub class. Shrub cells had a yearly probability of becoming tree covered, which increased over time (after the last fire disturbance) and depended on mature forest neighboring cells (ie. colonizer sources). Forests are considered mature when they have reached reproductive capacity to colonize shrub zones. The main tree species in the region reach maturity at about 15 years (*MatureForest*) after a stand replacing perturbation [25,27]. The probability of shrubland becoming forested was modeled as a logistic regression parameter (A6 in Appendix 1). Data used in the calibration was based on shrub cells from the first time window becoming forest or remaining stable in the 11-years period from 1989 to 2000. The intercept and slope of the linear predictor were *a* = -1.72 and *b* = 0.11 respectively. Since the model temporal scale is fixed and one time step represented one year, a yearly probability was calculated from the fitted 11-year probability. Ability of trees to colonize shrubland cells (*SeedPressure*) was two times higher for seeders than for resprouters [28].

**Literature Cited**

1. Ibàñez JJ, Burriel JA, Pons X (2002) El Mapa de Cobertes del Sòl de Catalunya: Una eina per al coneixement, la planificació i la gestió del territori. Perspectives territorials: 10–25.

2. Vallejo Bombín R (2005) El Mapa Forestal de España escala 1: 50.000 (MFE50) como base del tercer Inventario Forestal Nacional. Cuadernos de la Sociedad Española de Ciencias Forestales: 205–210.

3. Gil-Tena A, Saura S, Brotons L (2007) Effects of forest composition and structure on bird species richness in a Mediterranean context: Implications for forest ecosystem management. Forest Ecol Manag 242: 470–476.

4. Villanueva J (2004) Tercer Inventario Forestal Nacional (1997–2007). Madrid: Ministerio de Medio Ambiente y Medio Rural y Marino.

5. Gunnarsson F, Holm S, Holmgren P, Thuresson T (2012) On the potential of Kriging for forest management planning. Scandinavian Journal of Forest Research 13: 237–245. doi:doi: 10.1080/02827589809382981.

6. González JR, Pukkala T (2007) Characterization of forest fires in Catalonia (north-east Spain). Eur J Forest Res 126: 421–429. doi:10.1007/s10342-006-0164-0.

7. Diaz-Delgado R, Lloret F, Pons X (2004) Spatial patterns of fire occurrence in Catalonia, NE, Spain. Land Ecol 19: 731–745.

8. Catry FX, Rego FC, Bação FL, Moreira F (2009) Modeling and mapping wildfire ignition risk in Portugal. Int J Wildland Fire 18: 921. doi:10.1071/WF07123.

9. Burriel JA, Castro F, Mata T, Montserrat D, Gabriel de Francisco E, et al. (2007) La mejora del mapa diario de riesgo de incendio forestal en Cataluña. Barcelona: Departament de Medi Ambient. Generalitat de Catalunya.

10. Vallecillo S, Brotons L, Thuiller W (2009) Dangers of predicting bird species distributions in response to land-cover changes. Ecol Appl 19: 538–549.

11. Castellnou M, Miralles M, Pages J, Pique M (2009) Tipificación de los incendios forestales de Cataluña. Elaboración del mapa de incendios de diseño como herramienta para la gestión forestal. Proceedings of the 5th Congreso Forestal Español Ávila, Spain.

12. Ninyerola M, Pons X (2000) A methodological approach of climatological modelling of air temperature and precipitation through GIS techniques. Int J Climat: 1823–1841.

13. Piñol J, Terradas J, Lloret F (1998) Climate warming, wildfire hazard, and wildfire occurrence in coastal eastern Spain. Climatic Change: 345–357.

14. Podur JJ, Martell DL, Stanford D (2010) A compound poisson model for the annual area burned by forest fires in the province of Ontario. Environmetrics:. 21: 457–469.

15. Cui W, Perera AH (2008) What do we know about forest fire size distribution, and why is this knowledge useful for forest management? Int J Wildland Fire 17: 234. doi:10.1071/WF06145.

16. He HS, Mladenoff DJ (1999) Spatially explicit and stochastic simulation of forest-landscape fire disturbance and succession. Ecology 80: 81–99.

17. Rothermel R (1972) A mathematical model for predicting fire spread in wildland fuels. USDA Forest Service Research Paper INT USA.

18. Gonzalez-Olabarria JR, Pukkala T (2011) Integrating fire risk considerations in landscape-level forest planning. Forest Ecol Manag 261: 278–287. doi:10.1016/j.foreco.2010.10.017.

19. Millington JDA, Wainwright J, Perry GLW, Romero-Calcerrada R, Malamud BD (2009) Modelling Mediterranean landscape succession-disturbance dynamics: A landscape fire-succession model. Environ Model Softw 24: 1196–1208. doi:10.1016/j.envsoft.2009.03.013.

20. De Luis M, Baeza MJ, Raventós J, González-Hidalgo JC (2004) Fuel characteristics and fire behaviour in mature Mediterranean gorse shrublands. Int J Wildland Fire 13: 79–87.

21. González J, Palahi M, Trasobares A (2006) A fire probability model for forest stands in Catalonia (north-east Spain). Ann For Sci: 169–176.

22. Piñol J, Castellnou M, Beven K (2007) Conditioning uncertainty in ecological models: Assessing the impact of fire management strategies. Ecol Model 207: 34–44. doi:10.1016/j.ecolmodel.2007.03.020.

23. MAPA (1980) Mapa de cultivos y aprovechamientos a escala 1: 50.000. Madrid: MAPA (Ministerio de Agricultura, Pesca y Alimentación).

24. Rodrigo A, Retana J, Pico FX (2004) Direct regeneration is not the only response of Mediterranean forests to large fires. Ecology 85: 716–729.

25. Pausas JG, Carbó E, Neus Caturla R, Gil JM, Vallejo R (1999) Post-fire regeneration patterns in the eastern Iberian Peninsula. Acta Oecologica 20: 499–508. doi:10.1016/S1146-609X(00)86617-5.

26. Trabaud L, Michels C (1985) Recovery of burnt Pinus halepensis Mill. forests. II. Pine reconstruction after wildfire. Forest Ecol Manag: 167–179.

27. Tapias R, Gil L, Fuentes Utrilla P, Pardos JA (2001) Canopy seed banks in Mediterranean pines of south‐eastern Spain: a comparison between Pinus halepensis Mill., P. pinaster Ait., P. nigra Arn. and P. pinea L. Journal of Ecology 89: 629–638. doi:10.1046/j.1365-2745.2001.00575.x.

28. Verdú M (2000) Ecological and evolutionary differences between Mediterranean seeders and resprouters. Journal of Vegetation Science 11: 265–268.

**Table 1**. List of the land covers and dominant tree species included in the MEDFIRE land cover type map (*LCT*) classified according to vegetation type, response to fire and possibility of undergoing changes in state.

| **LCT** | **Vegetation type** | **Fire response** | **Changes** |
| --- | --- | --- | --- |
| *Pinus halepensis* | forest | burnable | dynamic |
| *Pinus nigra* |  |  |  |
| *Pinus pinea* |  |  |  |
| *Pinus sylvestris* |  |  |  |
| *Quercus suber* |  |  |  |
| *Quercus ilex* |  |  |  |
| Other conifers |  |  |  |
| Other *Quercus* |  |  |  |
| Other trees |  |  |  |
| Shrubland | non-forest |  |  |
| Cropland |  |  | static |
| Grassland |  |  |  |
| Rock |  | Non-burnable |  |
| Urban |  |  |  |
| Water |  |  |  |

**Table 2**. List of dynamic and static spatial state variables representing landscape context in MEDFIRE model. We include variable values or ranges, units for quantitative variables, and data sources from which such values are derived.

| **Variable (*model name*)** | **Type** | **Values or Rang** | **Source data** |
| --- | --- | --- | --- |
| Land cover type (*LCT*) | Dynamic | *Pinus halepensis*, *Pinus nigra*, *Pinus pinea*, *Pinus sylvestris*, *Quercus suber*, *Quercus il*ex, Other conifers, Other *Quercus*, Other threes, Shrub, Cropland, Grassland, Rock, Urban, Water | MFE50, NFI2, NFI3, LCMC, MCA |
| Time since last fire (*TSF*) |  | [1, 200] year | Wildfire perimeters (DMA, ICC) |
| Ignition probability (*ProbIgnition*) |  | [0, 1] | Wildfire perimeters (DMA, ICC), *LCT89*, Catalonia Digital Climate Atlas (CREAF), Basic fire risk map and road network (DMA) |
| Bioclimatic region (*BioRegion*) | Static | North-West (NW), North-East (NE), South-Central (SC) | Vallecillo et al. 2009 |
| Fire spread type (*SpreadType*) |  | [0, 100] % | Castellnou et al. 2009 |
| Elevation (*Elevation*) |  | [-5, 3102] meter | Digital Elevation Model (ICC) |
| Aspect (*Aspect*) |  | North, South, East or West | Derived from Elevation |
| Main wind direction (*Wind*) |  | [0, 359] degree | Meteorological station records (DMA) |

**Table 3**. Description of all *Fire* sub-model parameters and data sources used for their initialization.

| **Process** | **Variable** | **Value** | **Description** | **Source data** |
| --- | --- | --- | --- | --- |
| Fire regime | *AnnualBurnDistNorm*  *AnnualBurnDistSevr*  *FireSizeDistNorm*  *FireSizeDistSevr* | μ=8.61 σ=0.82  μ=9.64 σ=0.73  α=3.15 β=0.72  α=3.18 β=0.68 | Distribution of annual area to burn for normal and severe years (~ log normal)  Distribution of fire sizes for normal and severe years (~ power law) | Calibration from historical fire statistics |
| Fire spread | *rWind*  *rSlope*  *rAspect*  *rTSF*  *rLCT*  *f_LCT_* | 0.9  0.9  0.3  0.3  0.6  Table 5 | Wind weight in fire spread rate (SR)  Slope weight in fire SR  Aspect weight in fire SR  TSF weight in fire SR  LCT flammability weight in fire SR  LCT flammability | Calibration from historical fire perimeters |
| Burning | *SR_BurnExp* | 0.9 | Exponent defining probability of burning as function of spread rate | Calibration from historical fire perimeters |
| Fire suppression | *SRthreshFF*  *TSFthreshFF* | In %  In years | Active fire suppression thresholds | Scenario parameter |

**Table 4**. Flammability fire-spread parameter (relative weights used in the fire spread algorithm) of burnable land cover types.

| **LCT** | ***f_LCT_*** |
| --- | --- |
| *Pinus halepensis* | 0.8 |
| *Pinus nigra* | 0.8 |
| *Pinus pinea* | 0.8 |
| *Pinus sylvestris* | 0.8 |
| *Quercus suber* | 0.4 |
| *Quercus ilex* | 0.4 |
| Other conifers | 0.8 |
| Other *Quercus* | 0.4 |
| Other trees | 0.4 |
| Shrub | 1 |
| Cropland | 0.15 |
| Grassland | 0.1 |

**Table 5**. Initialization and description of vegetation dynamics sub-model parameters.

| **Process** | **Variable** | **Value** | **Description** | **Consulted literature** |
| --- | --- | --- | --- | --- |
| Post-fire vegetation regeneration | *pNeighbContag*  *postFireSucc*  *postFireSuccReburnt*  *CanopySeedAge*  *SppAspectFactor* | 0.4    Table 7  Table 7  15  1:10 | Probability of spatially autocorrelated post-fire transitions  Post-fire transitions probabilities and adapted probabilities to recent reburnt areas  Years after fire disturbance to *Pinus halepensis* generates reproductive canopy seed bank  Ratio increasing the probability to turn on shrub in southern slopes after fire | Calibration  Rodrigo et al. (2004)  Tapias et al. (2001)  Pausas et al. (1999) |
| Succession from shrubland to forest | *MatureForest*  *a, b*  *SeedPressure* | 15  -1.72, 0.11  2 | Reproductive age of the main tree species in the study area  Intercept and slope of the linear prediction of logit (*P_shrub2forest_*)  Weight to represent the colonization capacity of seeder tree species | Pausas (1999)  Calibration  Verdú (2000) |

**Table 6**. Basic post-fire transition probabilities for dynamic land cover types (Table 1): dominant tree species and shrubland (in %). The probability of forest species to remain the same or to change to another species after fire are rescaled from Rodrigo et al. (2004) for the monospecific stands.

| **Pre-fire \ Post-fire** | **(1)** | **(2)** | **(3)** | **(4)** | **(5)** | **(6)** | **(7)** | **(8)** | **(9)** | **(10)** |
| --- | --- | --- | --- | --- | --- | --- | --- | --- | --- | --- |
| **(1)  *Pinus halepensis*** | 82 | 0 | 0 | 0 | 0 | 5 | 0 | 5 | 0 | 8 |
| **(2) *Pinus nigra*** | 2 | 0 | 0 | 0 | 0 | 23 | 0 | 27 | 0 | 48 |
| **(3)  *Pinus pinea*** | 0 | 0 | 2 | 0 | 12 | 0 | 9 | 0 | 0 | 77 |
| **(4) *Pinus sylvestris*** | 0 | 0 | 0 | 0 | 0 | 13 | 0 | 67 | 0 | 20 |
| **(5)  *Quercus suber*** | 0 | 0 | 0 | 0 | 99 | 0 | 0 | 0 | 0 | 1 |
| **(6) *Quercus ilex*** | 3 | 0 | 0 | 0 | 0 | 71 | 0 | 6 | 0 | 20 |
| **(7) Other conifers** | 0 | 0 | 0 | 0 | 10 | 0 | 73 | 0 | 0 | 17 |
| **(8) Other *Quercus*** | 0 | 0 | 0 | 0 | 0 | 3 | 0 | 93 | 0 | 4 |
| **(9) Other trees** | 0 | 0 | 0 | 0 | 0 | 0 | 0 | 0 | 100 | 0 |
| **(10) Shrubland** | 0 | 0 | 0 | 0 | 0 | 0 | 0 | 0 | 0 | 100 |

**Figure 1**. Distribution of the mean percentage of area burnt per fire for the five *LCT*-clusters drawn for the study area (A) and the three bioclimatic regions: North-East (B), North-West (C) and South-Central (D). Forest tree species were merged and so burnable covers define five new target *LCT*-clusters: **P** - *Pinus halepensis*, *Pinus nigra*, *Pinus pinea*, *Pinus sylvestris* and Other conifers; **Q** - *Quercus suber*, *Quercus ilex*, and Other *Quercus*; **O** - Other trees; **S** – Shrub; and **C** - Cropland. Distributions represent 100 MEDFIRE replicates for a subset of 117 historical fires for which fire spread was simulated from known ignition locations. Solid black squares show for each *LCT*-cluster the mean percentage of observed area burnt per fire for the historical fire data subset used.


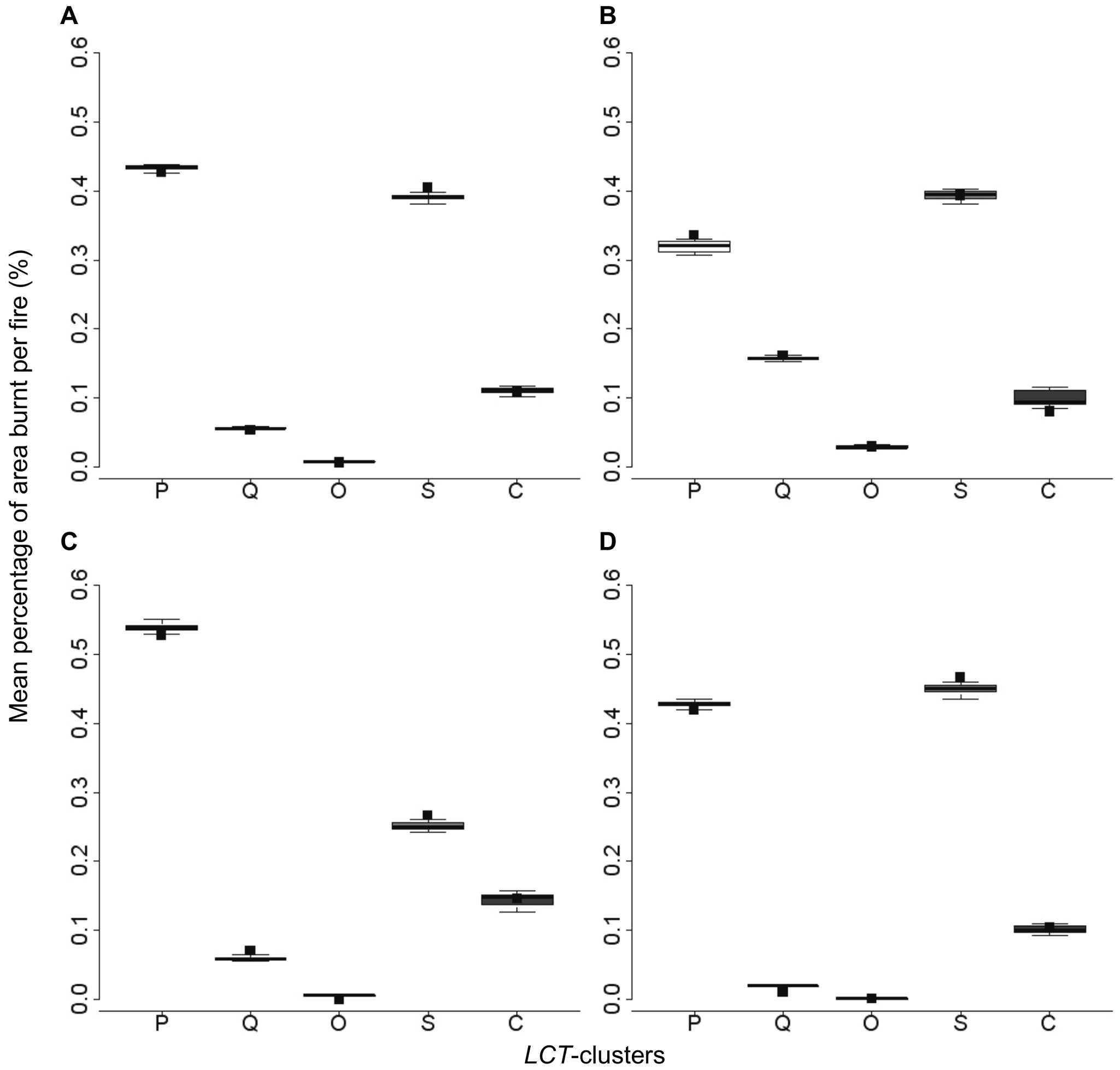

Supplement: Appendix S2 — Initialization of model variables. This appendix describes the main data sources employed to initialize spatial state variables and parameters used in the MEDFIRE model and described in Appendix S1. (DOC) [file pone.0062392.s004.doc]
